# Genome‐wide analysis of in vivo CcpA binding with and without its key co‐factor HPr in the major human pathogen group A *Streptococcus*

**DOI:** 10.1111/mmi.14667

**Published:** 2020-12-29

**Authors:** Sruti DebRoy, Victor Aliaga‐Tobar, Gabriel Galvez, Srishtee Arora, Xiaowen Liang, Nicola Horstmann, Vinicius Maracaja‐Coutinho, Mauricio Latorre, Magnus Hook, Anthony R. Flores, Samuel A. Shelburne

**Affiliations:** ^1^ Department of Infectious Diseases Infection Control and Employee Health University of Texas MD Anderson Cancer Center Houston TX USA; ^2^ Facultad de Ciencias Químicas y Farmacéuticas Advanced Center for Chronic Diseases‐ACCDiS Universidad de Chile Independencia Chile; ^3^ Laboratorio de Bioingeniería Instituto de Ciencias de la Ingeniería Universidad de O'Higgins Rancagua Chile; ^4^ Center for Infectious and Inflammatory Diseases Institute of Biosciences and Technology Texas A&M University Health Science Center Houston TX USA; ^5^ Centro de Modelamiento Molecular, Biofísica y Bioinformática (CM2B2) Facultad de Ciencias Químicas y Farmacéuticas Universidad de Chile Santiago Chile; ^6^ Laboratorio de Bioinformática y Expresión Génica INTA Universidad de Chile Santiago Chile; ^7^ Mathomics Center for Mathematical Modeling Universidad de Chile Santiago Chile; ^8^ Center for Genome Regulation (Fondap 15090007) Universidad de Chile Santiago Chile; ^9^ Division of Infectious Diseases Department of Pediatrics University of Texas Health Science Center McGovern Medical School Houston TX USA; ^10^ Center for Antimicrobial Resistance and Microbial Genomics University of Texas Health Science Center McGovern Medical School Houston TX USA; ^11^ Department of Genomic Medicine University of Texas MD Anderson Cancer Center Houston TX USA; ^12^ Present address: Department of Experimental Therapeutics University of Texas MD Anderson Cancer Center Houston TX USA

**Keywords:** ChIP‐seq, HPr‐independent CcpA regulation, *Streptococcus pyogenes*

## Abstract

Catabolite control protein A (CcpA) is a master regulator of carbon source utilization and contributes to the virulence of numerous medically important Gram‐positive bacteria. Most functional assessments of CcpA, including interaction with its key co‐factor HPr, have been performed in nonpathogenic bacteria. In this study we aimed to identify the in vivo DNA binding profile of CcpA and assess the extent to which HPr is required for CcpA‐mediated regulation and DNA binding in the major human pathogen group A *Streptococcus* (GAS). Using a combination RNAseq/ChIP‐seq approach, we found that CcpA affects transcript levels of 514 of 1667 GAS genes (31%) whereas direct DNA binding was identified for 105 GAS genes. Three of the directly regulated genes encode the key GAS virulence factors Streptolysin S, PrtS (IL‐8 degrading proteinase), and SpeB (cysteine protease). Mutating CcpA Val301 to Ala (strain 2221‐CcpA‐V301A) abolished interaction between CcpA and HPr and impacted the transcript levels of 205 genes (40%) in the total CcpA regulon. By ChIP‐seq analysis, CcpAV301A bound to DNA from 74% of genes bound by wild‐type CcpA, but generally with lower affinity. These data delineate the direct CcpA regulon and clarify the HPr‐dependent and independent activities of CcpA in a key pathogenic bacterium.

## Graphical abstract

The carbon catabolite protein, CcpA, regulates carbohydrate utilization and virulence in concert with its phosphorylated co‐factor histine containing protein (HPr~P). In this study we engineered a CcpA isoform that does not interact with HPr~P. Using a combination of ChIPseq and RNAseq we report the in vivo binding sites of CcpA in group A Streptococcus. We also demonstrate that CcpA retains the ability to bind and regulate genes even when it cannot interact with HPr~P.

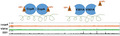

## 1 INTRODUCTION

Carbon catabolite repression (CCR) is a global process by which bacteria prioritize the use of favorable energy sources (Deutscher et al., 2006; Fujita, 2009). The core mechanism of CCR is an alteration in levels of proteins involved in metabolite transport and utilization which in turn is primarily achieved at the transcriptional level (Gorke & Stulke, 2008). The LacI‐GalR family transcriptional regulator catabolite control protein (CcpA) is a key mediator of CCR in many Gram‐positive bacteria (Henkin et al., 1991; Titgemeyer & Hillen, 2002; Warner & Lolkema, 2003). CcpA inactivation impacts ~15%–20% of the transcriptome of a broad array of Gram‐positive bacteria with the majority of impacted genes encoding proteins involved in carbohydrate and nitrogen utilization (Antunes et al., 2012; DebRoy et al., 2016; Seidl et al., 2009; Zeng et al., 2013). Importantly, CcpA also affects the transcript levels of genes encoding known and putative virulence factors in human Gram‐positive pathogens ranging from streptococci to clostridia (Iyer et al., 2005; Mertins et al., 2007; Seidl, Bischoff, et al., 2008; Varga et al., 2004). Consequently, CcpA inactivation in diverse organisms result in altered virulence‐related phenotypes such as extracellular capsule production, biofilm formation, lysis of red blood cells, and inter‐species signaling (Giammarinaro & Paton, 2002; Johnson et al., 2009; Kinkel & McIver, 2008; Seidl, Goerke, et al., 2008; Watson et al., 2013).

Although CcpA is clearly necessary to the virulence of numerous key Gram‐positive pathogens, understanding of CcpA physiologic function is mainly derived from studies in nonpathogenic bacteria (Warner & Lolkema, 2003). Based primarily on investigations in *Bacillus* species, CcpA is currently thought to impact gene expression by binding *cis*‐acting DNA known as catabolite response elements (*cre*) which are composed of the pseudo‐palindromic motif WTGNAANCGNWNNCWW (where W = A or T and N = any base) (Miwa et al., 2000; Schumacher et al., 2011; Stulke & Hillen, 2000). CcpA affinity for *cre* sites is significantly increased by the co‐effector molecule, histidine‐containing protein (HPr) phosphorylated at Ser46 (HPrSer46~P) (Aung‐Hilbrich et al., 2002; Deutscher et al., 2005; Shelburne et al., 2008). HPr is phosphorylated and dephosphorylated at Ser46 by HPr kinase/phosphorylase (HPrK/P), a bifunctional ATP‐dependent enzyme whose activity is responsive to intracellular energy status (Poncet et al., 2004). Thus, the interaction of CcpA with HPrSer46~P facilitates alteration in gene expression in response to metabolic changes (Deutscher et al., 2006). All major, invasive Gram‐positive pathogens contain highly conserved orthologs of CcpA, HPr, and HPrK/P with amino acid similarities ranging from 70% for CcpA to 85% for HPr. However, *Bacillus* species also contain Crh (catabolite repression HPr), which is an HPr‐like protein important for CcpA‐mediated gene regulation that is not present in typical Gram‐positive pathogens such as staphylococci and streptococci (Deutscher et al., 2006; Galinier et al., 1997; Schumacher et al., 2006).

Although the pseudo‐palindromic *cre* sequence seems to be the predominate site of CcpA‐DNA interaction in *Bacillus* species, there have been suggestions that CcpA may bind other DNA sites. Using in silico approaches, *cre* sites have been predicted in only a small percentage of genes whose transcript levels are significantly altered by CcpA inactivation (Carvalho et al., 2011; DebRoy et al., 2016). Additionally, a recent study in *Clostridium acetobutylicum* identified a CcpA binding motif (TGTAA/TTTACA) which is quite distinct from the previous *cre* motif (Yang et al., 2017). Moreover, in addition to the “classic” *cre* motif, a genome‐wide CcpA binding study identified a *cre*2 motif in *Streptococcus suis* (TTTTYHWDHHWWTTTY, where Y is C or T, H is A or C or T, and D is A or G or T) that was primarily important for CcpA function in the stationary phase (Willenborg et al., 2014). Although CcpA transcriptome analyses have been performed in a wide variety of bacteria, genome‐wide characterization of CcpA‐DNA binding is not currently available for a major, invasive Gram‐positive pathogen other than *Streptococcus suis* (Antunes et al., 2012; Buescher et al., 2012; Willenborg et al., 2014).

In addition to a sub‐optimal understanding of how CcpA interacts with DNA in invasive Gram‐positive bacteria, analysis of the role of HPr and HPrK/P in pathogenic bacteria has been quite limited (Mertins et al., 2007; Shelburne et al., 2008). In part, this is because in major Gram‐positive pathogens such as group A *Streptococcus* (GAS), *Streptococcus pneumoniae*, and *Staphylococcus aureus*, HPr or even HPrSer46~P appears to be essential, in contrast to what is observed in *Bacillus* species (Fleming et al., 2015). In *Staphylococcus xylosu*s elimination of HPrSer46~P completely abolished CCR suggesting that CcpA absolutely requires HPrSer46~P to impact gene transcription (Jankovic & Bruckner, 2002). In contrast, CcpA regulation at *cre*2 sites in *S. suis* was postulated to be independent of HPr (Willenborg et al., 2014). As pathogens establish and propagate human infections, they are likely to encounter vastly different metabolic conditions, which in turn would be expected to alter HPrSer46~P levels. Thus, establishing whether CcpA can function independently of HPrSer46~P and which genes are regulated by CcpA in the absence of HPrSer46~P is necessary to fully understand the role that nutritional acquisition plays in modulation of pathogenesis and infection outcomes. Herein, we sought to determine the global DNA binding characteristics of CcpA in GAS and analyze the effect of blocking the interaction between CcpA and HPrSer46~P in order to broaden insight into the physiology underlying the critical contribution of CcpA to Gram‐positive pathogenesis.

## 2 RESULTS

### 2.1 A CcpA mutant that does not bind HPr

We chose to conduct our study in the *emm1* GAS strain MGAS2221 because *emm1* strains are leading causes of GAS infections, MGAS2221 is representative of the current *emm1* strains causing human disease worldwide, and MGAS2221 has a fully sequenced genome and lacks mutations in known regulators that have been shown to impact the CcpA transcriptome (Shelburne et al., 2010; Sumby et al., 2005). Both HPr and HPrK/P are essential in GAS (Le Breton et al., 2015). Thus, to probe into the possible existence of a CcpA regulon independent of HPrSer46~P, we first attempted to generate an HPr‐S46A mutant. However, we were unable to generate a viable GAS HPr‐S46A mutant despite repeated efforts suggesting that, as in the case of *S. pneumoniae* (Fleming et al., 2015), a S46A mutation in GAS HPr is lethal, possibly due to HPrSer46~P dependent cellular functions that are essential. We also made several attempts to generate a HPrK‐D197A mutant which would prevent Ser46 phosphorylation of HPr, but this mutation was also not viable. As an alternate approach we sought to generate a GAS mutant which retains the ability for HPr to be phosphorylated, but does not permit interactions between HPrSer46~P and CcpA. In silico alanine scanning mutagenesis of the CcpA‐HPrSer46~P interface, modeled on the crystal structure of the *Bacillus megaterium* CcpA‐HPrSer46~P‐DNA complex, identified four amino acid residues of CcpA that are critical to CcpA‐HPrSer46~P interaction (Homeyer et al., 2007; Schumacher et al., 2004). We chose to alter one of these residues, valine 301, to alanine given that the V301 residue is conserved in all CcpA family proteins (Schumacher et al., 2004) and gene regulation studies have demonstrated that a CcpAV301 mutant is compromised in glucose‐mediated gene repression (Sprehe et al., 2007).

**FIGURE 1:**
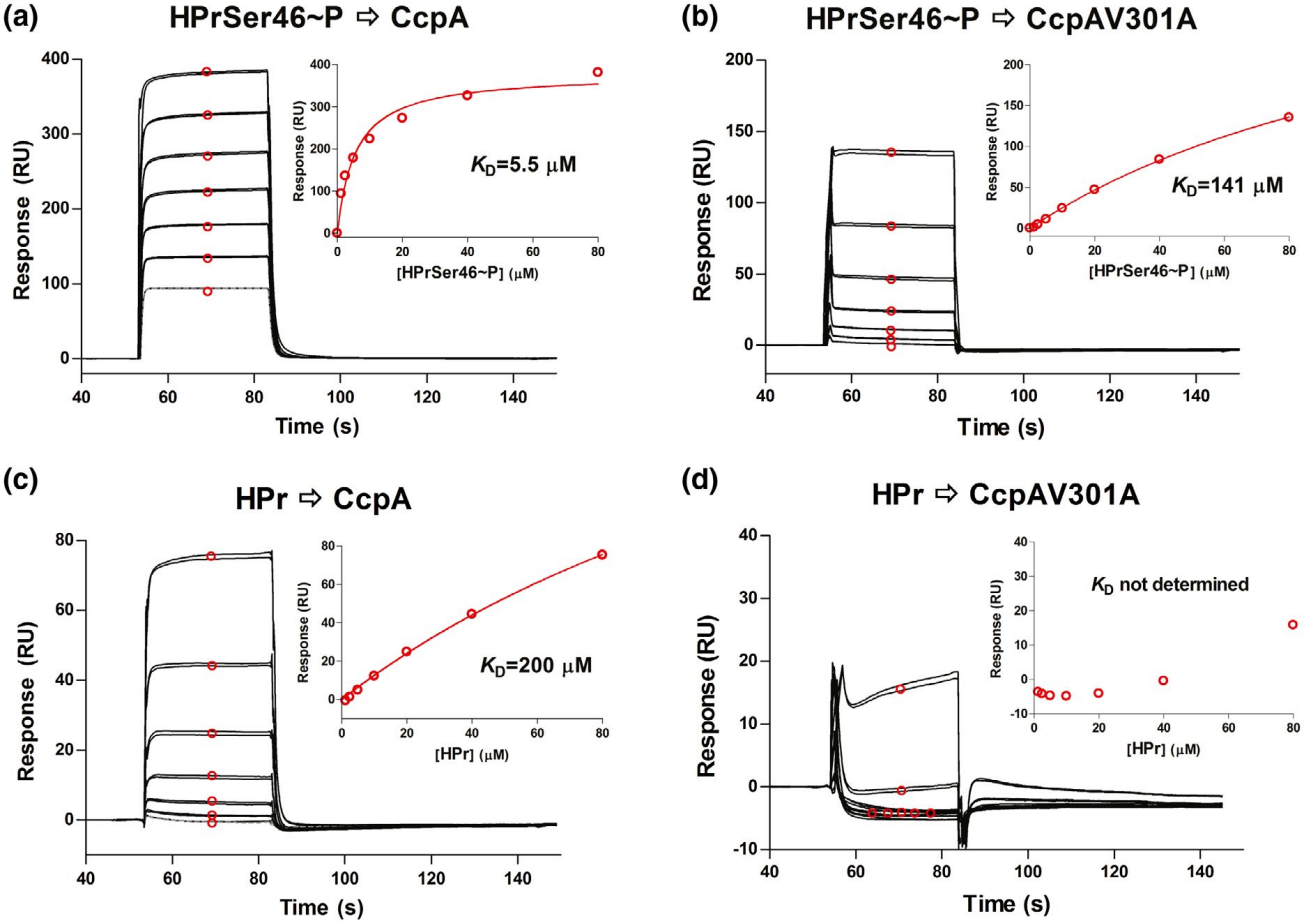
In vitro analysis of CcpA‐HPr interaction. Representative SPR analysis (*n* = 2) of the binding between CcpA and HPr recombinant proteins. HPrSer46~P and HPr (2‐fold serial dilutions from 1.25 to 80 μM) was injected in duplicate to (a & c) CcpA surface (3,700 RU) and (b & d) CcpAV301A surface (4,200 RU). The SPR response curves of bound protein are shown in black with lower curve corresponding to lower concentration of protein injected. The average responses at steady state (shown in red circles) were plotted as a function of the HPr concentration and the isotherm was fit to a one‐site binding (hyperbola) model (fitted curve shown in red) to determine equilibrium dissociation constant *K*_D_ (inset)

We first sought to test the hypothesis that the CcpAV301A mutation would impair the interaction of recombinant CcpA and HPr using surface plasmon resonance (SPR). To this end, we expressed and purified recombinant CcpA and CcpAV301A along with HPr. We used recombinant HPrK/P to phosphorylate HPr at Ser46 as previously described (Shelburne et al., 2010). As expected, wild type CcpA had about 30‐fold higher affinity (i.e., bound more tightly) for HPrSer46~P relative to HPr with *K*_D_ value of 5.8 ± 0.5 versus 188 ± 18 µM (Figure 1). Consistent with our hypothesis that V301 is critical for CcpA‐HPrSer46~P interaction in GAS, recombinant CcpAV301A bound to HPrSer46~P with a *K*_D_ value of 153 ± 17 µM which closely approximated that observed for wild type CcpA/HPr interaction (*K*_D_ = 188 ± 18 µM)(Figure 1). The interaction between CcpAV301A and HPr is much weaker and the affinity could not be determined under the conditions used. The equilibrium dissociation constant values (*K*_D_) provided in the text are mean ± standard deviation obtained from two experiments while those in Figure 1 are from a representative experiment.

**FIGURE 2:**
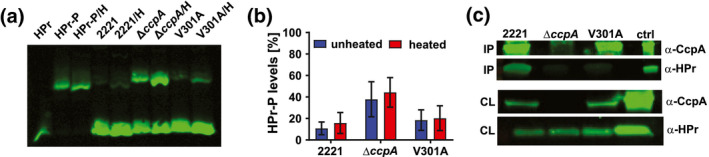
Characterization of the CcpAV301A mutant protein. (a) Representative Phostag Western blot (*n* = 2) and (b) graphical representation of the levels of phosphorylated HPr in lysates of wild type (2221), *ccpA* deletion mutant (∆*ccpA*) and the CcpAV301A mutant (V301A) grown to mid‐exponential phase. Lanes marked with “H” indicate heated samples. Purified recombinant HPr and HPr~P was used as controls. Error bars indicate standard deviation. (c) Co‐immunoprecipitation of HPr from lysates of indicated strains using anti‐CcpA antibody. “IP” and “CL” indicates immunoprecipitated material and cell lysate, respectively. The antibody for each sub‐panel is indicated on the right. Purified recombinant CcpA and HPr proteins were used as controls (ctrl)

Next, we used site directed mutagenesis to create the isoallellic mutant strain 2221‐CcpA‐V301A in the same parental background, the serotype *emm1* strain MGAS2221, as our previously created 2221∆*ccpA* isolate (Table 1). Given that deleting *ccpA* has previously been shown to affect HPr/HPr~P ratios in other bacteria (Leboeuf et al., 2000; Ludwig et al., 2002), we used Phos‐tag gels to analyze HPr and HPr~P levels at the mid‐exponential phase of growth in nutrient rich Todd‐Hewitt media (0.2% glucose present in THY activates the HPr kinase and subsequent phosphorylation of HPrSer46~P). Under these conditions, the majority of HPr in strains MGAS2221 and 2221‐CcpA‐V301A was unphosphorylated with no significant difference of HPr/HPr~P ratios identified between the two strains (Figure 2a,b). Conversely, HPr~P levels were increased in strain 2221∆*ccpA* relative to the other two strains. A similar increase in HPr~P levels has been observed in *ccpA* mutants of *B. subtilis* and *E. faecalis*, and has been attributed to increased HPr kinase activity (Leboeuf et al., 2000; Ludwig et al., 2002). These data show that the V301A alteration in CcpA did not have significant effects on cellular HPr~P levels (Figure 2a,b). Additionally, the cellular levels of CcpA were similar between MGAS2221 and 2221‐CcpA‐V301A indicating that the amino acid variation did not impact CcpA autoregulation (note similar CcpA band densities for MGAS2221 and 2221‐CcpA‐V301A in Figure 2c, third panel).

**TABLE 1:** Strains and plasmids used in this study

Strain or plasmid	Description	Reference
*Strains*		
MGAS2221	Invasive clinical isolate, reference serotype M1	Sumby et al. (2006)
2221Δ*ccpA*	MGAS2221 Δ*ccpA*::*spc*	Shelburne et al. (2010)
2221*‐*CcpA‐V301A	MGAS2221 with V301A change in CcpA	This study
BL21‐pET‐His2‐CcpA	*E. coli* producing recombinant wild type GAS CcpA	Shelburne et al. (2008)
BL21‐pET‐His2‐V301A	*E. coli* producing recombinant GAS CcpA with V301A change	This study
BL21‐pET‐His2‐HPr	*E. coli* producing recombinant GAS HPr	Shelburne et al. (2010)
BL21‐pET21a‐HPrKP	*E. coli* producing recombinant GAS HPrK/P	Shelburne et al. (2010)
*Plasmids*		
pET‐His2‐CcpA	pET‐His2 plasmid with GAS *ccpA* gene	Shelburne et al. (2008)
pET‐His2‐V301A	pET‐His2 plasmid with GAS *ccpA* gene encoding V301A change	This study
pET‐His2‐HPr	pET‐His2 plasmid with GAS HPr gene	Shelburne et al. (2010)
pET21a‐HPrK/P	pET21a plasmid with GAS HPrK/P gene	Shelburne et al. (2010)
pBBL740 *ccpA*V301A	pBBL740 plasmid with GAS *ccpA* gene encoding V301A change	This study

To further test our hypothesis that CcpAV301A was not interacting with HPr~P in vivo, we performed immunoprecipitation reactions using anti‐CcpA antibody which detects both the wild type and CcpAV301A mutant protein (Figure 2c, first and third panels). Strains MGAS2221, 2221Δ*ccpA*, and 2221‐CcpA‐V301A were cross‐linked and harvested at mid‐exponential phase. CcpA‐containing complexes were immunoprecipitated using a polyclonal anti‐CcpA antibody, and then, analyzed for the presence of HPr by western blotting using anti‐HPr antibody (Figure 2c). By quantitative analysis, 10 times more HPr was immunoprecipitated by CcpA antibody from strain MGAS2221 compared to strain 2221‐CcpA‐V301A. No HPr was immunoprecipitated by CcpA antibody in strain 2221Δ*ccpA*. Taken together, we conclude that the V301A amino acid change essentially abolishes the ability of CcpA to interact with HPr~P both in vitro and in vivo.

### 2.2 Inability of CcpA to interact with HPr~P impacts the CcpA transcriptome

**FIGURE 3:**
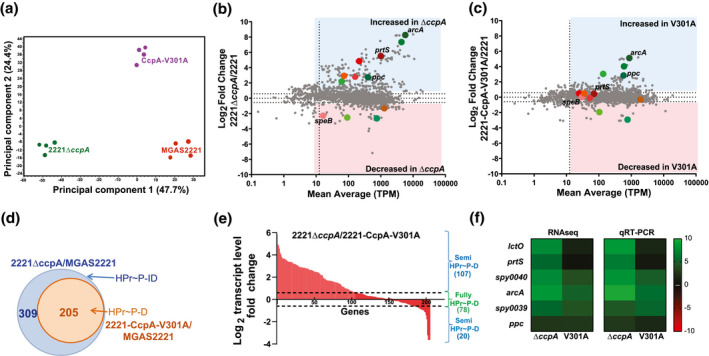
Impact of CcpAV301A mutation on the GAS transcriptome. (a) Principal component analysis showing that the transcriptomes of MGAS2221, 2221∆*ccpA* and 2221‐CcpA‐V301A are distinct. Each strain has four biological replicates. MA plots displaying transcriptome comparisons of (b) *ccpA* deletion (∆*ccpA*/MGAS2221) and (c) isoallelic CcpA V301A mutant (2221‐CcpA‐V301A/MGAS2221) to wild type MGAS2221. Colored quadrants include transcripts that are differentially regulated in each comparison as per the criteria outlined in the text. Select genes are color coded to indicate if they are HPr~P‐dependent (shades of green) or HPr~P‐independent (shades of red). (d) Venn diagram showing the subsets of *ccpA*‐affected genes that are HPr~P‐independent (HPr‐ID) and HPr~P‐dependent (HPr‐D). The strain comparisons used to generate these subsets are indicated in their respective colors. (e) Waterfall plot showing the gradation in the magnitude of the transcriptional impact of the CcpAV301A mutation on the HPr~P‐dependent genes. The fold change for HPr~P‐dependent genes between strains 2221∆*ccpA* and 2221‐CcpA‐V301A, as observed in the RNAseq data, is plotted. The genes within the dotted lines are fully HPr~P‐dependent, while those outside are HPr~P semi‐dependent. The number of genes in each category are specified in parentheses. (f) Gene transcript levels of selected CcpA‐impacted genes that exhibit varying transcriptional effects of the CcpAV301A mutation by RNAseq analysis were validated by targeted Taqman qRT‐PCR analysis. Transcript levels in strains 2221∆*ccpA* and 2221‐CcpA‐V301A are shown relative to wild type MGAS2221. For Taqman qRT‐PCR, data are mean ± standard deviation of two biological replicates, with two technical replicates, done on two separate days (*n* = 8)

Given that CcpA‐HPrSer46~P interaction is thought to be critical for CcpA activity (Deutscher, 2008; Deutscher et al., 2005, 2006; Gorke & Stulke, 2008; Stulke & Hillen, 2000), we next sought to test the hypothesis that the CcpAV301A change would result in a transcriptome similar to that observed for a complete CcpA knockout. To this end, we performed RNAseq analyses, in quadruplicate, for strains MGAS2221, 2221Δ*ccpA*, and 2221‐CcpA‐V301A grown to mid‐exponential phase in THY. The RNAseq data described herein is new and is not that reported in our previously published study (DebRoy et al., 2016). We did not observe any significant changes in *ccpA* transcript levels between MGAS2221 and 2221‐CcpA‐V301A, confirming that the V301A change does not affect transcription of *ccpA* itself (Figure S1). Principal component analysis showed that the data were reproducible and, contrary to our hypothesis, that the transcriptomes of the three strains were quite distinct (Figure 3a). We assigned transcript levels as being significantly different when there was a mean difference of at least 1.5‐fold and a *p* < .05 after accounting for multiple comparisons. We first sought to assess how the transcriptome of MGAS2221differed from 2221Δ*ccpA*. Deletion of *ccpA* resulted in significantly different transcript levels of 514 genes representing ~31% of the 1667 genes in the MGAS2221 genome with sufficient transcript levels for analysis (Figure 3b; Table S1). More genes (361) had increased transcript levels in strain 2221∆*ccpA* (i.e., were CcpA repressed) compared to genes (153) whose transcript levels were lower in 2221∆*ccpA* (i.e., were CcpA activated), which is in accordance with previous observations (Carvalho et al., 2011; DebRoy et al., 2016; Kietzman & Caparon, 2011; Shelburne et al., 2008). The transcript levels of 12 virulence factor encoding genes were impacted by CcpA inactivation including *speB*, *speA2*, sic, *spd*, *ska*, *grab*, the streptolysin S (*sag*) operon, the *nga*‐*slo* operon, and *prtS* (Table 2). Consistent with the central role of CcpA in carbon source acquisition and utilization, the transcript levels of genes encoding 13/14 of the phosphotransferase systems (PTS) present in MGAS2221 were significantly altered by CcpA inactivation as were genes encoding four ATP binding cassette (ABC) carbohydrate transporters (Table 3). Cluster of orthologous group (COG) analysis revealed that genes whose transcript levels were significantly impacted by CcpA inactivation were more likely to be in groups C (energy production and conversion) and G (carbohydrate transport and metabolism) relative to unaffected genes (Figure S2a,b).

**TABLE 2:** Influence of CcpA‐HPr~P interaction on virulence gene regulation and DNA binding

M5005 spy#	Gene	CcpA repressed vs. activated	HPr~P dependent vs. independent	If HPr~P dependent : semi or fully	Bound by CcpA	Bound by CcpAV301A
0139‐0141	*nga‐slo*	Repressed	Dependent (*nga*)	Semi	No	No
0341	*prtS*	Repressed	Independent		Yes	Yes
0351	*spyA*	Repressed	Independent		No	No
0562‐70	Streptolysin S operon	Repressed	Dependent	Semi	Yes	Yes
0996	*speA2*	Repressed	Independent		No	No
1106	*grab*	Activated	Dependent	Semi	No	No
1540	*endoS*	Activated	Dependent	Semi	No	No
1684	*ska*	Repressed	Independent		No	No
1711	*lmb*	Repressed	Independent		No	No
1718	*sic*	Repressed	Independent		No	No
1735	*speB*	Activated	Independent		Yes	Yes
1738	*spd*	Repressed	Independent		No	No

**TABLE 3:** Influence of CcpA‐HPr~P interaction on regulation and DNA binding of genes encoding carbohydrate transport systems of MGAS2221

M5005 spy#	Putative transported carbohydrate	Transporter type	CcpA repressed vs. activated	HPr~P dependent vs. independent	Bound by CcpA	Bound by CcpAV301A
0212‐0219	Sialic acid	ABC	Repressed	Independent	Yes	No
0475	Β‐glucoside	PTS	Repressed	Semi‐dependent	No	No
0521	N‐acetylglucosamine	PTS	Repressed	Independent	No	No
0662	Fructose	PTS	Repressed	Fully ‐dependent	No	No
0780‐0783	Mannose/fructose	PTS	Repressed	Semi‐dependent	No	No
1058‐1060	Maltose	ABC	Repressed	Semi‐dependent	Yes	Yes
1067‐1062	Maltodextrin	ABC	Repressed	Semi‐dependent	Yes	Yes
1083‐1079	Cellobiose	PTS	Repressed	Semi‐dependent	Yes	Yes
1310‐1308	Unknown sugar	ABC	Repressed	Semi‐dependent	No	No
1399‐1401	Galactose	PTS	Repressed	Semi‐dependent	No	No
1479‐1481	Mannose	PTS	Repressed	Semi‐dependent	Yes	Yes
1542	Sucrose	PTS	Activated	Semi‐dependent	No	No
1634‐1633	Lactose	PTS	Activated	Fully ‐dependent	No	No
1664‐1662	Mannitol	PTS	Repressed	Semi‐dependent	No	No
1692	Maltose	PTS	Repressed	Independent	Yes	Yes
1746‐1744	Cellobiose	PTS	Repressed	Semi‐dependent	Yes	No
1784	Trehalose	PTS	Repressed	Semi‐Dependent	No	No

Abbreviations: ABC, ATP binding cassette; PTS, phosphotransferase system;

Next, we compared the transcriptomes of 2221‐CcpA‐V301A and MGAS 2221 (Figure 3c) and analyzed transcript levels in strain 2221‐CcpA‐V301 for genes whose transcript levels were significantly different between strains MGAS2221 and 2221Δ*ccpA*. If the CcpA‐HPr~P interaction is critical to regulation of a particular CcpA‐impacted gene then we would expect that transcript levels for said gene to be significantly different between strains MGAS221 and 2221‐CcpA‐V301A (e.g., compare *arcA* and *ppc* between Figure 3b,c). However, of the 514 genes whose transcript levels were significantly affected by CcpA inactivation, only 205 (40%) also had significantly different transcript levels in strain 2221‐CcpA‐V301A relative to strain MGAS2221 (Figure 3d). We considered these to be HPr~P‐dependent genes because eliminating the interaction between CcpA and HPr~P impacted their transcript levels (Table S1). The remaining 309 (60%) genes whose transcript levels were impacted by CcpA inactivation did not have significantly different transcript levels between strains 2221‐CcpA‐V301A and MGAS2221 (e.g., compare *prtS* and *speB* between Figure 3b,c), and were, therefore, denoted as HPr~P‐independent (Figure 3d) (Table S1). A significantly higher percentage of the HPr~P‐dependent genes were CcpA repressed (157/205, 77%) compared to HPr~P‐independent genes (204/309, 66%, *p* = .01 by Fisher's exact test). For the 12 virulence factor encoding genes/operons impacted by CcpA inactivation, only four were HPr~P‐dependent (*nga*‐*slo*, *sag* operon, *grab*, and *endoS*). Conversely, of the 17 CcpA‐regulated genes/operons encoding carbohydrate transport systems, 14 were HPr~P‐dependent (Tables 2 and 3). Similarly, COG analysis showed that, the HPr~P‐dependent genes were more likely to be in category G (carbohydrate transport and metabolism) and less likely to be in category L (replication and repair), *M* (cell wall/membrane/envelop biogenesis), and S (function unknown) relative to HPr~P‐independent genes (Figure S2c,d).

We next sought to determine whether the loss of CcpA‐HPr~P interaction was equivalent to complete CcpA inactivation in terms of the absolute effect on gene expression by using fold‐change (FC) comparison of the HPr~P‐dependent genes. If the CcpA‐HPr~P complex is essential for CcpA‐regulation of a particular gene, then, there should be no significant difference in transcript levels between strains 2221Δ*ccpA* and 2221‐CcpA‐V301A. In fact, similar transcript levels in these two strains were observed for only 78 (38%) of the 205 HPr~P‐dependent genes, which we subsequently considered as fully HPr~P‐dependent (i.e., the effect of eliminating CcpA‐HPr~P interaction was equivalent to the effect of total CcpA inactivation) (Table S1). For the remaining 127/205 (62%) genes, transcript levels were significantly different between strains 2221∆*ccpA* and 2221‐CcpA‐V301A. Given that CcpA is primarily a repressor, for these 127 genes the loss of interaction with HPr~P resulted in transcript levels that were generally lower than complete CcpA inactivation, but the magnitude of effect varied for individual genes (Figure 3e). We designated these genes as semi‐HPr~P‐dependent (Table S1). All four HPr~P‐dependent virulence factor encoding genes/operons (*nga*‐*slo* operon, *sag* operon, *grab*, and *endoS*) were semi‐HPr~P‐dependent (Table 2). Of the 14 HPr~P‐dependent carbohydrate utilization encoding operons, only two (lactose and fructose transporters) were fully HPr~P‐dependent (Table 3). We chose CcpA‐regulated genes that exhibit varying degrees of transcriptional impact upon disrupting the CcpA‐HPr~P interaction and verified the changes in transcript levels using targeted qRT‐PCR (Figure 3f). Taken together, our data show that CcpA influences a significant proportion of the GAS genome even without HPr~P interaction and that the quantitative impact of HPr~P on CcpA‐mediated regulation is distinct for different genes.

### 2.3 In vivo interaction of CcpA with GAS chromatin

There are limited genome wide assessments of CcpA binding to DNA in pathogenic bacteria and none for a strain in which CcpA‐HPr~P interaction has been abrogated (Willenborg et al., 2014). Therefore, we performed ChIP‐seq analysis for strains MGAS2221 and 2221‐CcpA‐V301A grown to mid‐exponential phase in THY media to analyze CcpA chromatin occupancy profiles and assess how the reduced ability of CcpA to interact with HPr~P affects CcpA‐DNA binding. The 2221∆*ccpA* strain was used as a control to ensure the specificity of our findings.

**FIGURE 4:**
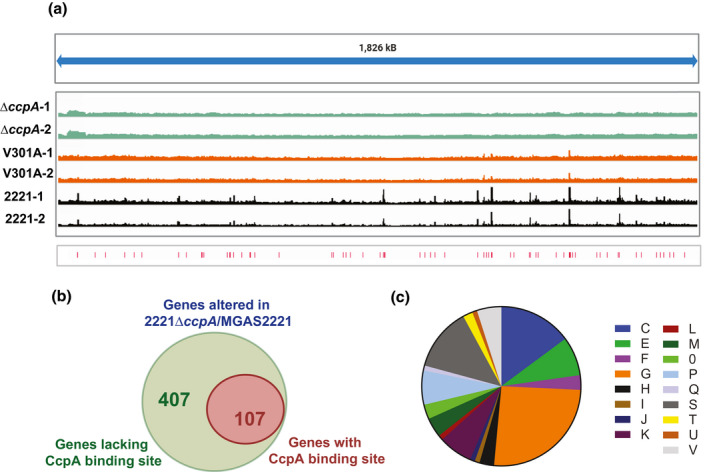
Characterization of in vivo DNA binding of CcpA. (a) Linear representation of indicated strains showing the CcpA binding sites as determined by ChIP‐seq analysis. Replicates of each strain analyzed are shown and peak positions are indicated by tally marks in the bottom sub‐panel. (b) Venn diagram showing proportion of genes identified as CcpA‐regulated by RNAseq that have CcpA binding sites as identified in our ChIP‐seq analysis. (c) COG distribution of genes that have CcpA binding sites and are transcriptionally altered in 2221∆*ccpA*. [C] Energy production and conversion; [D] Cell cycle control, cell division, chromosome partitioning; [E] Amino acid transport and metabolism; [F] Nucleotide transport and metabolism; [G] Carbohydrate transport and metabolism; [H] Coenzyme transport and metabolism; [I] Lipid transport and metabolism; [J] Translation, ribosomal structure and biogenesis; [K] Transcription; [L] Replication, recombination and repair; [M] Cell wall/membrane/envelope biogenesis; [N] Cell motility; [O] Posttranslational modification, protein turnover, chaperones; [P] Inorganic ion transport and metabolism; [Q] Secondary metabolites biosynthesis, transport, and catabolism; [S] Function unknown; [T] Signal transduction mechanisms; [U] Intracellular trafficking, secretion, and vesicular transport and [V]Defense mechanisms

We identified 76 CcpA binding sites in strain MGAS2221, including in the promoter region of *ccpA* itself which is consistent with a previous in vitro analysis of CcpA binding (Almengor et al., 2007) (Table 4). The genome‐wide distribution of these sites is shown in Figure 4a. The lack of significant enrichment at any of these sites in strain 2221∆*ccpA* indicates that these are true in vivo binding sites for the CcpA protein. The CcpA binding sites were mostly (67%) in predicted promoter regions (i.e., within 500 bps of a translational start site) (Fujita, 2009), which is in accord with other CcpA ChIP‐seq analyses (Antunes et al., 2012; Buescher et al., 2012; Willenborg et al., 2014). Of the 76 enriched regions, 46 sites (61%) were associated with genes whose transcript levels were significantly altered in strain 2221∆*ccpA* relative to strain MGAS221. These 46 sites were associated with 45 operons containing 107 genes. The discrepancy between numbers of binding sites and operons affected is due to the occasional identification of multiple distinct binding sites for the same gene. Thus, only 107 (21%) of the 514 genes whose transcript levels were significantly impacted by CcpA inactivation in strain MGA2221were identified as being directly regulated by CcpA (Figure 4b). The vast majority of sites enriched in the ChIP‐seq analysis (88%) correlated with genes which had increased transcript levels in strain 2221∆*ccpA* relative to MGAS2221 (i.e., were CcpA‐repressed). By COG analysis, genes directly regulated by CcpA were significantly more likely to be in category C (energy production and conversion) and G (carbohydrate transport and metabolism) (Figure 4c).

**TABLE 4:** CcpA binding sites identified by ChIP‐seq analysis and associated genes

CBS^a^	Bound by WT	Bound by V301A	*cre*	*cre* location^b^	*cre* sequence	Locus tag^c^	Gene	Annotation	COG	Regulated by CcpA	HPr‐dependency
1	Yes	Yes	Yes	Prom	AAAGAAAGCGGTTTCA	M5005_Spy_0039	*adh2*	Bifunctional acetaldehyde‐CoA/alcohol dehydrogenase	C	Yes	Semi dependent
2	Yes	Yes	Yes	Prom	ACAGAAAACGATTTCA	M5005_Spy_0094	*ackA*	Acetate kinase	F	Yes	Independent
3	Yes	No	Yes	ORF	ATGGTGGCGTTTTCTT	M5005_Spy_0128	*ntpE*	V‐type ATP synthase subunit E	C	Yes	Independent
4	Yes	Yes	Yes	Prom	AACGAAAACCTTTTCA	M5005_Spy_0180	–	Hypothetical protein	D	No	NA
4	Yes	Yes	Yes	Prom	AACGAAAACCTTTTCA	M5005_Spy_0185	*pgi*	pgi	G	No	NA
5	Yes	No	Yes	Prom	TTGAAAGCGCTTTATT	M5005_Spy_0212	–	N‐acetylmannosamine‐6‐phosphate 2‐epimerase	G	Yes	Independent
6	Yes	Yes	Yes	Prom	AAAGAAAGCCCTTTCC	M5005_Spy_0233	*plr*	Aldehyde dehydrogenase	G	No	NA
7	Yes	Yes	Yes	Prom	AATGTAAGCGCTAACAAAAT	M5005_Spy_0339	*exoA*	Exodeoxyribonuclease	L	Yes	Independent
7	Yes	Yes	Yes	Prom	AATGTAAGCGCTAACAAAAT	M5005_Spy_0340	*lctO*	L‐lactate oxidase	C	Yes	Semi dependent
8	Yes	Yes	Yes	ORF	TAGGAAGCGTTTTCTT	M5005_Spy_0341	*prtS*	Peptidase S8	O	Yes	Independent
9	Yes	No	Yes	ORF	GTGCAAGCGCTTTGAT	M5005_Spy_0362	*gcaD*	Glucosamine‐1‐phosphate acetyltransferase	M	No	NA
10	Yes	No	No	–	–	M5005_Spy_0416		Glutamine cyclotransferase	O	No	NA
10	Yes	No	No	–	–	M5005_Spy_0417	*pcp*	Pyrrolidone‐carboxylate peptidase	O	No	NA
11	Yes	No	No	–	–	M5005_Spy_0417	*pcp*	Pyrrolidone‐carboxylate peptidase	O	No	NA
12	Yes	No	Yes	Prom	TTGAAAACTTTTTCAA	M5005_Spy_0424	*ccpA*	Catabolite control protein A	K	Yes	Independent
13	Yes	No	No	–	–	M5005_Spy_0495	*lysS*	Lysine‐‐tRNA ligase	J	No	NA
13	Yes	No	No	–	–	M5005_Spy_0496		Haloacid dehalogenase	S	No	NA
14	Yes	No	No	–	–	M5005_Spy_0504	*pepF*	Oligoendopeptidase F	E	No	NA
15	Yes	Yes	Yes	ORF	TTTGGGAACGATTTCTCAAG	M5005_Spy_0771	–	CRISPR‐associated endonuclease Cas2	L	No	NA
16	Yes	Yes	Yes	Prom	AAATAAAGCGCTTACT	M5005_Spy_0505	*ppc*	Phosphoenolpyruvate carboxylase	H	Yes	Fully dependent
17	Yes	Yes	Yes	Prom	GAGAAAACGTTTTAGT	M5005_Spy_0512	–	Sugar phosphate phosphatase	S	Yes	Fully dependent
18	Yes	No	Yes	Prom	TTGACACCGTTTTCAT	M5005_Spy_0533	–	Hypothetical protein	S	Yes	Independent
18	Yes	No	Yes	Prom	TTGACACCGTTTTCAT	M5005_Spy_0534	–	Acetoin reductase	IQ	Yes	Semi dependent
19	Yes	No	Yes	ORF	GAAGATATCGCTTCTA	M5005_Spy_0556	*eno*	Phosphopyruvate hydratase	F	No	NA
20	Yes	No	Yes	Prom	TATTATATCGATTTCT	M5005_Spy_0555	–	Hypothetical protein	S	No	NA
20	Yes	No	Yes	Prom	TATTATATCGATTTCT	M5005_Spy_0556	*eno*	Phosphopyruvate hydratase	F	No	NA
21	Yes	Yes	Yes	Prom	AAGAAAGGGTTTACAT	M5005_Spy_0562	*sagA*	Streptolysin S family bacteriocin		Yes	Semi dependent
22	Yes	No	Yes	ORF	ATGGAAGCTTTTTCAG	M5005_Spy_0622	–	Alkaline phosphatase	M	No	NA
22	Yes	No	Yes	ORF	ATGGAAGCTTTTTCAG	M5005_Spy_0625	*aroF*	Chorismate synthase	E	No	NA
23	Yes	No	Yes	ORF	GTGAAGGGTTTATCAT	M5005_Spy_0772	–	Type II‐A CRISPR‐associated protein Csn2	S	No	NA
24	Yes	No	No	–	–	M5005_Spy_0778	*msrB*	Peptide‐methionine (R)‐S‐oxide reductase	O	No	NA
25	Yes	Yes	Yes	ORF	GAAGATAACGATTTCA	M5005_Spy_0817	*dacA1*	D‐alanyl‐D‐alanine carboxypeptidase	M	No	NA
26	Yes	No	Yes	ORF	TTGTAAGCGCTACCGA	M5005_Spy_0823	*folQ*	Dihydroneopterin aldolase	H	No	NA
27	Yes	Yes	Yes	Prom	AAGAAAGGGTTTTCAA	M5005_Spy_0834	–	Alcohol dehydrogenase	E	Yes	Semi dependent
27	Yes	Yes	Yes	Prom	AAGAAAGGGTTTTCAA	M5005_Spy_0835	–	Acid phosphatase/phosphotransferase	S	Yes	Semi dependent
28	Yes	No	Yes	Prom	ACTGATAACGCTTCCAA	M5005_Spy_0873	*ldh*	L‐lactate dehydrogenase	C	No	NA
28	Yes	No	Yes	Prom	ACTGATAACGCTTCCAA	M5005_Spy_0874	*gyrA*	DNA gyrase subunit A	L	No	NA
29	Yes	No	No	–	–	M5005_Spy_0925	*rnhB*	Hypothetical protein	F	No	NA
30	Yes	Yes	Yes	Prom	CTTGAAACCGCTTGCT	M5005_Spy_0934	*‐*	Lipoate‐‐protein ligase	H	Yes	Fully dependent
31	Yes	No	Yes	Prom	AATGAAAGCGTTTATA	M5005_Spy_0938	*pgmA*	Phosphoglucomutase	G	Yes	Semi dependent
32	Yes	No	No	–	–	M5005_Spy_0938	*pgmA*	Phosphoglucomutase	G	Yes	Semi dependent
33	Yes	No	No	–	–	M5005_Spy_0938	*pgmA*	Phosphoglucomutase	G	Yes	Semi dependent
34	Yes	Yes	Yes	ORF	TAAGATACCGCTTGCA	M5005_Spy_1055	*glgP*	Maltodextrin phosphorylase	G	Yes	Independent
34	Yes	Yes	Yes	ORF	TAAGATACCGCTTGCA	M5005_Spy_1058	*malE*	Maltose/maltodextrin‐binding protein	G	Yes	Independent
35	Yes	Yes	Yes	Prom	CTGCAAGCGGTTGCAT	M5005_Spy_1057	*malR*	LacI family transcriptional regulator	K	Yes	Independent
35	Yes	Yes	Yes	Prom	CTGCAAGCGGTTGCAT	M5005_Spy_1058	*malE*	Maltose/maltodextrin‐binding protein	G	Yes	Independent
36	Yes	Yes	Yes	Prom	ATCGTAATCGCTTTCA	M5005_Spy_1067	*malX*	Sugar ABC transporter substrate‐binding protein	G	Yes	Semi dependent
37	Yes	Yes	Yes	Prom	TTAGAAAACGCTTTCT	M5005_Spy_1083	*bglG*	Transcription antiterminator BglG	G	Yes	Semi dependent
38	Yes	Yes	Yes	ORF	CTAAAAGCGTTTTCTC	M5005_Spy_1096	–	Thioesterase	Q	No	NA
39	Yes	No	Yes	Prom	CATGATAACCCTTACA	M5005_Spy_1122	*nrdH*	NrdH‐redoxin	O	Yes	Independent
39	Yes	No	Yes	Prom	CATGATAACCCTTACA	M5005_Spy_1121	*ptsH*	Phosphocarrier protein HPr	G	Yes	NA
40	Yes	Yes	Yes	Prom	CAAGAAATCGCTTTCT	M5005_Spy_1235	–	Phosphomannomutase	G	Yes	Semi dependent
41	Yes	No	Yes	ORF	CAGAAAACTCTTTCTT	M5005_Spy_1250	*ftsA*	Cell division protein FtsA	D	No	NA
42	Yes	No	Yes	Prom	ATGGAATCGCTTTCTA	M5005_Spy_1258	–	Hypothetical protein	S	Yes	Independent
43	Yes	No	Yes	ORF	ATCGTAAGCGCCTCCA	M5005_Spy_1265	–	Ribose operon repressor		No	NA
44	Yes	No	Yes	ORF	GTAAAATCTTTTTCTG	M5005_Spy_1272	–	Arginine:ornithine antiporter	S	Yes	Semi dependent
45	Yes	No	No	–	–	M5005_Spy_1274		N‐acetyltransferase	K	Yes	Semi dependent
46	Yes	No	No	–	–	M5005_Spy_1274		N‐acetyltransferase	K	Yes	Semi dependent
47	Yes	No	No	–	–	M5005_Spy_1274		N‐acetyltransferase	K	Yes	Semi dependent
48	Yes	Yes	Yes	Prom	TGAGTAATCGCTTACA	M5005_Spy_1275	*arcA*	Arginine deiminase	E	Yes	Semi dependent
48	Yes	Yes	Yes	Prom	TGAGTAATCGCTTACA	M5005_Spy_1277	*ahrC.2*	Arginine regulator	K	Yes	Semi dependent
49	Yes	Yes	Yes	ORF	CTGCAATCGTTTACTT	M5005_Spy_1319	–	RNA methyltransferase	J	No	NA
49	Yes	Yes	Yes	ORF	CTGCAATCGTTTACTT	M5005_Spy_1323	–	Hypothetical protein	L	No	NA
50	Yes	Yes	Yes	Prom	CTTGAAGCGCTTACTT	M5005_Spy_1328	–	YigZ family protein	S	Yes	Independent
50	Yes	Yes	Yes	Prom	CTTGAAGCGCTTACTT	M5005_Spy_1325	–	Ribosome‐associated factor Y	J	Yes	Fully dependent
51	Yes	No	No	–	–	M5005_Spy_1366		Penicillin‐binding protein 2X	M	No	NA
52	Yes	Yes	Yes	ORF	GAGAAAAGGATTTCAT	M5005_Spy_1367	*ftsL*	Cell division protein FtsL	D	No	NA
53	Yes	Yes	Yes	Prom	AAGTAAGCGTTTTCCT	M5005_Spy_1381	*glpK*	Glycerol kinase	F	Yes	Independent
54	Yes	No	No	–	–	M5005_Spy_1382		Hypothetical protein	T	Yes	Independent
55	Yes	Yes	Yes	Prom	CTGTAAGCGATTACTT	M5005_Spy_1387	–	2,5‐diketo‐D‐gluconic acid reductase	C	Yes	Independent
56	Yes	No	No	–	–	M5005_Spy_1448		Nuclease	S	No	NA
57	Yes	Yes	Yes	Prom	GTGAAAACGTTTTAAA	M5005_Spy_1477	–	NCS2 family permease	S	Yes	Semi dependent
57	Yes	Yes	Yes	Prom	GTGAAAACGTTTTAAA	M5005_Spy_1479	*manL*	PTS mannose transporter subunit EIIAB	G	Yes	Semi dependent
58	Yes	Yes	Yes	Prom	AAAGAAAACGTTTTCT	M5005_Spy_1496	*phaB*	Enoyl‐CoA hydratase	I	Yes	Independent
59	Yes	No	No	–	–	M5005_Spy_1497	*dnaJ*	Chaperone DnaJ	O	Yes	Fully dependent
60	Yes	No	Yes	Prom	AAAGAAAACACTTGCA	M5005_Spy_1503	*–*	Histidine phosphatase family protein	G	Yes	Independent
61	Yes	No	No	–	–	M5005_Spy_1513		Aminotransferase	E	No	NA
61	Yes	No	No	–	–	M5005_Spy_1514		Universal stress protein UspA	T	No	NA
62	Yes	Yes	Yes	Prom	TGGGAAAACGTTTCCT	M5005_Spy_1569	*pfl*	Formate acetyltransferase	C	Yes	Independent
63	Yes	No	No	–	–	M5005_Spy_1575	*norA*	MFS transporter	EGP	Yes	Fully dependent
64	Yes	No	Yes	Prom	TTTAAAGCTTTTTAA	M5005_Spy_1599	*pgk*	Phosphoglycerate kinase	F	No	NA
65	Yes	Yes	Yes	Prom	ATAAAAGCGTTATCTC	M5005_Spy_1624	*–*	Hypothetical protein		No	NA
66	Yes	Yes	Yes	ORF	AGAGAAACCGGTACCA	M5005_Spy_1627	*salY*	ABC transporter permease	V	No	NA
67	Yes	Yes	Yes	Prom	TGCGCAAGCGCTTGCA	M5005_Spy_1680	*pulA*	Pullulanase	G	No	NA
68	Yes	Yes	Yes	Prom	GATGCAATCGCTTGCA	M5005_Spy_1692	–	PTS maltose	G	Yes	Independent
69	Yes	Yes	Yes	ORF	GTGATAGCGCTATCTT	M5005_Spy_1734	*speB*	Streptopain	M	Yes	Independent
69	Yes	Yes	Yes	ORF	GTGATAGCGCTATCTT	M5005_Spy_1736	–	Hypothetical protein		Yes	Independent
70	Yes	No	Yes	Prom	TTGTAATCGTTTACAT	M5005_Spy_1746	–	PTS cellobiose transporter subunit IIA	G	Yes	Semi dependent
71	Yes	No	No	–	–	M5005_Spy_1758		Dipeptidase	M	Yes	Semi dependent
72	Yes	Yes	Yes	Prom	GTGAAAGCGTTATCGT	M5005_Spy_1758	–	Dipeptidase	M	Yes	Semi dependent
73	Yes	Yes	Yes	Prom	ATGTAAGCGTTATCTAA	M5005_Spy_1772	–	Glutamate formimidoyltransferase	E	Yes	Independent
73	Yes	Yes	Yes	Prom	ATGTAAGCGTTATCTAA	M5005_Spy_1770	*hutI*	Imidazolonepropionase	Q	Yes	Semi dependent
74	Yes	Yes	Yes	Prom	CATGAAAACGCCTCCA	M5005_Spy_1779	*rpsB*	ATP‐binding protein	T	Yes	Semi dependent
75	Yes	No	No	–	–	M5005_Spy_1807	*argR2*	Arginine repressor	K	Yes	Independent
76	Yes	Yes	Yes	ORF	ACAGATAACGCTTACT	M5005_Spy_1865	*htrA*	Serine protease	O	No	NA

Abbreviation: NA, not applicable.

^a^ CcpA binding site number

^b^ Prom, binding site located in noncoding promoter region upstream of ATG. ORF, binding site identified within coding region of gene.

^c^ Locus tag numbers are based on the MGAS5005 genome.

**FIGURE 5:**
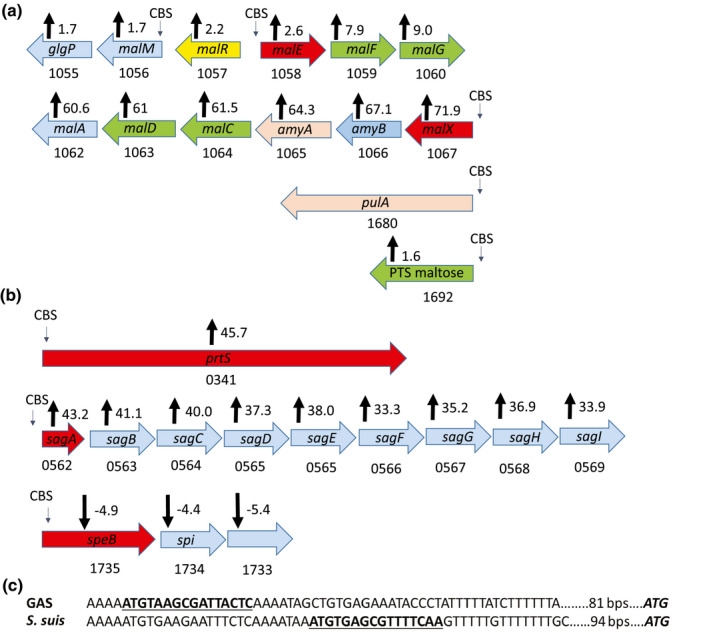
CcpA‐bound sites in MGAS2221. Schematic representation of the binding sites for CcpA (CBS) in key (a) carbohydrate transport and (b) virulence factor encoding genes. Fold change in transcript levels upon CcpA inactivation is displayed with black arrows. Positive numbers indicate higher transcript levels in 2221∆*ccpA* compared to MGAS2221. Genes in the schematic diagram are color coded to indicate function: blue—intracellular carbohydrate processing protein; yellow—transcriptional regulator; red—substrate binding lipoprotein; green—transport protein and beige—cell surface/secreted protein. (c) Sequence variation in the promoter region of the arginine deiminase (*arcA*) gene in GAS and *S. suis*. Enriched site for GAS and putative CcpA binding site for *S. suis* are highlighted

Specific CcpA binding was identified for genes encoding four PTS systems known and putatively involved in the transport and utilization of maltose, cellobiose (two operons), and mannose, which stands in contrast to the 13 PTS gene/operons whose transcript levels were affected by CcpA inactivation. We also observed CcpA binding sites in genes/operons encoding three ABC carbohydrate transport systems for maltose, maltodextrin, and sialic acid. The central role of maltose/maltodextrin utilization in GAS pathophysiology is reflected by the five distinct CcpA binding sites for genes involved in the acquisition of these prevalent carbohydrates including that in the promoter region of the *pulA* gene encoding a cell‐surface pullulanase important for both nutrient acquisition and adherence (Figure 5a). In addition to transporters, genes encoding glycerol kinase, phosphoglucomutase, and HPr, which are also involved in carbohydrate metabolism, had CcpA binding sites and were directly regulated by CcpA.

CcpA enriched sites were also identified in the promoters of genes encoding various amino acid uptake and utilization pathways such as those involved in arginine, histidine, and glutamate metabolism. Although a recent CcpA study in *Streptococcus suis* did not identify in vivo CcpA binding for *arcA* (Willenborg et al., 2014), the initial gene in the arginine catabolism operon, *arcA* showed the strongest CcpA‐mediated DNA enrichment for the entire MGAS2221 data set (Figure S3). A comparison of the *arcA* promoters showed that whereas a CcpA binding site is present in GAS and predicted for *S. suis*, these sites are quite variable both in terms of composition and location, which may explain the divergent results (Figure 5c).

Importantly, DNA enrichment was observed for the promoter and/or 5’ coding regions of three key GAS virulence factor encoding genes (Figure 5b). Namely we observed in vivo binding of CcpA for *sagA*, the first gene in the operon encoding the key cytotoxin Streptolysin S, *speB*, which encodes an actively secreted broad‐spectrum cysteine proteinase, and *prtS* which encodes an IL‐8 degrading enzyme. No CcpA binding or effect on gene transcript level was observed for *cfa* (M5005_spy0981) which was previously reported to be directly regulated by CcpA (Kietzman & Caparon, 2010). Similarly, we did not observe enrichment for the other nine virulence factor encoding genes whose transcript levels were varied by CcpA inactivation (Table 2) suggesting an indirect mechanism for CcpA impact on these genes.

Given the large numbers of genes impacted by CcpA inactivation relative to the number of identified CcpA binding sites, it was reasonable to suspect that many of the genes might be secondarily affected by regulators under CcpA control. Indeed, RNAseq analysis showed that the transcript levels of 21 genes/operons encoding known and putative transcriptional regulators, including four two‐component gene regulatory systems (TCS), were significantly affected by CcpA inactivation (Table S1). Of these, however, we identified direct CcpA binding for only three genes encoding the transcriptional regulators BglG, ArgR2, and Spy1495 (Table 4). While the *bglG* and *argR2* genes had a CcpA‐binding site in their promoters, *spy1495* was in an operon where the first gene (*spy1496*) was CcpA‐bound. As previously noted, there was also CcpA enrichment in the intergenic region between *malE* and *malR* (Figure 5a), which encodes a maltose regulatory protein. Hence, *malR* could also be directly regulated by CcpA although the location of the binding site suggests that CcpA is more likely to influence *malE* transcription. There was no DNA enrichment for any TCS or the master regulator Mga (multigene activator), which was identified to be directly regulated by CcpA in a previous study (Almengor et al., 2007). Given that none of the regulators directly controlled by CcpA are known or expected to have large transcriptomes (Shelburne et al., 2011), these data suggest that the broad impact of CcpA inactivation on GAS gene transcript levels is unlikely to be primarily mediated by regulators directly under CcpA control.

### 2.4 Analysis of CcpAV301A in vivo DNA Binding

Of the 76 DNA loci bound by CcpA, 37 enriched regions which encompass 75 genes (12% of the CcpA transcriptome) were also enriched in strain 2221‐CcpA‐V301A (Figure 4a). All of the enriched sites in 2221‐CcpA‐V301 were associated with genes that also evidenced enriched sites in MGAS2221. All three of the virulence factor encoding genes bound by CcpA in strain MG22221, *prtS*, *sagA*, and *speB*, also evidenced binding in strain 2221‐CcpA‐V301A (Tables 2 and 4). Similarly, five of the seven PTS/ABC carbohydrate transport encoding genes/operons (Tables 3 and 4) bound by CcpA were also bound by CcpAV301A as was *arcA* (Table 4). In contrast, *spy1746*, which encodes the first gene in a cellobiose PTS operon, and *spy0212*, which encodes the first gene in the sialic acid uptake ABC operon were not bound by CcpAV301A. We also did not observe binding of the CcpAV301A protein to the promoter of either *ccpA*, *ptsH* (HPr) or the operon encoding the ATP synthase genes (Table 4). When considering all DNA binding sites for the CcpA protein in strain MGAS2221, those sites that were also bound in strain 2221‐CcpA‐V301A were more likely to have significantly different transcript levels following CcpA inactivation (*p* < .001 by Fisher's exact test). To validate the findings from our ChIP‐seq, six sites that were enriched in both the wild type MGAS2221 and the 2221‐CcpA‐V301A strain were confirmed by SYBR qRT PCR (Figure 7a).

### 2.5 Characterization of CcpA binding sites not associated with differentially expressed genes

For 29 CcpA binding sites, we did not observe a significant difference in transcript levels for a nearby gene following CcpA inactivation. The majority of these binding sites were located in the middle of genes suggesting that the observed CcpA binding is likely nonfunctional. For nine genes, the DNA enrichment site overlapped with promoter regions including four genes whose function would suggest regulation by CcpA (Table 4). These four genes were *pgi*, which encodes a glucose utilization protein, *plr*, which encodes an aldehyde dehydrogenase, *ldh*, which encodes an enzyme that converts lactate to pyruvate, and the previously mentioned *pulA*. Of these 29 CcpA enriched sites that were not associated with transcript level variation after CcpA inactivation, 11 were also enriched in strain 2221‐CcpA‐V301 including three genes encoding carbohydrate utilization proteins (*pgi*, *plr*, and *pulA*) as well as one site in a CRISPR operon and the *salY* gene, which encodes an ABC transport protein involved in lantibiotic secretion (Table 4). The combination of CcpA binding site location and putative function suggests that these genes may be directly regulated by CcpA under conditions distinct from those studied herein.

### 2.6 Identification of CcpA binding motif

**FIGURE 6:**
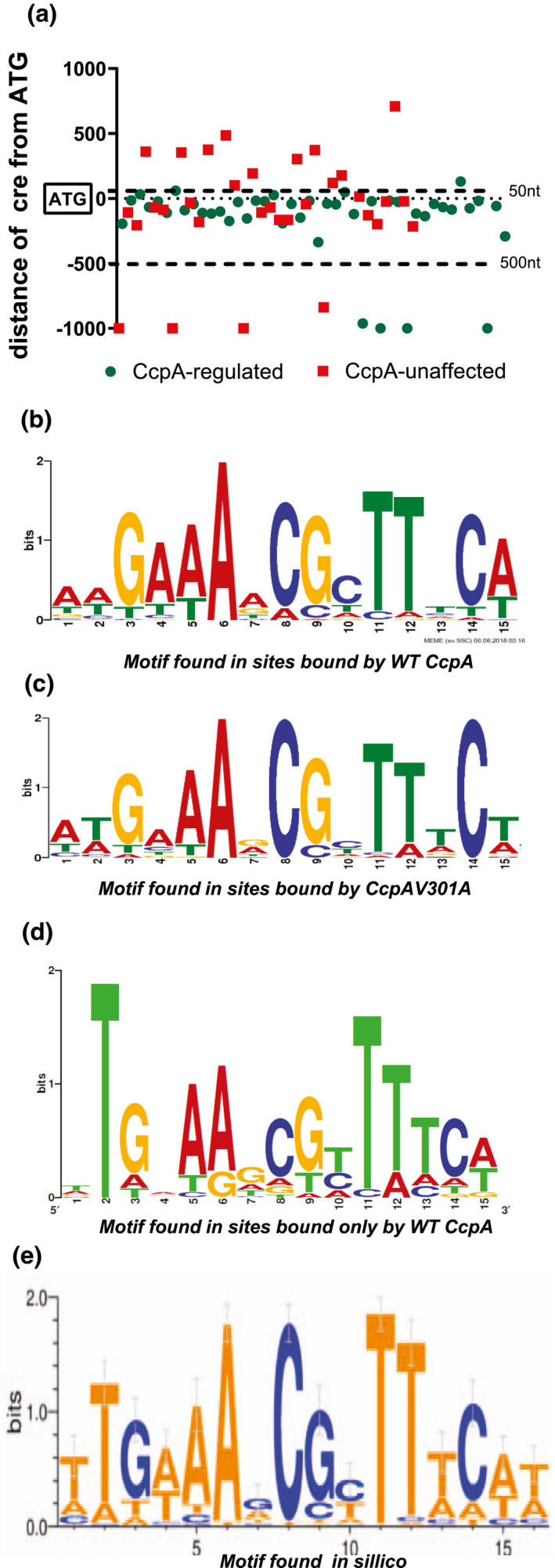
Consensus motifs identified in CcpA binding sites. (a) Scatter plot showing the distance from the translational start site (TSS) of enriched sites bound by the wild type CcpA protein that contain a consensus motif. Genes associated with the enriched sites are color coded to indicate whether they are (CcpA‐regulated) or not (CcpA‐unaffected) transcriptionally impacted upon CcpA inactivation. Enriched sites that are located farther than 1,000 nt from the TSS are not plotted. WebLogo representation of the consensus *cre* motif identified from CcpA binding sites in strains (b) MGAS2221 and (c) 2221‐CcpA‐V301A. (d) The *cre* motif found in sites that were bound by the wild type CcpA but not the CcpAV301A mutant. (e) The consensus motif identified in our previous study by in silico analysis of CcpA‐regulated genes in three different GAS serotypes (DebRoy et al., 2016)

Using the ChIP‐seq data, we identified a single consensus motif in the enriched sites for MGAS2221 (Figure 6b) which was highly consistent with previously identified *cre* motifs from *Bacillus* species (Marciniak et al., 2012; Schumacher et al., 2004). This motif was present in 57 of the 76 CcpA enriched sites (75%). For the remaining 19 sites, no particular consensus motif was identified. We did not identify the *cre*2 sequence of *S. suis* or the flexible binding site of *Clostridium acetobutylicum* that were recently described in any of the enriched sites in MGAS2221 (Willenborg et al., 2014; Yang et al., 2017). Compared to enriched areas with *cre* sites, enriched areas lacking *cre* sites were less likely to be in genes/gene promoters whose transcript levels were significantly affected by CcpA inactivation (*p* = .01 by Fisher's exact test), suggesting that the *cre*‐containing enriched DNA sites were more biologically impactful under the studied conditions. The *cre* sites that were associated with genes whose transcript levels were affected by CcpA inactivation, were significantly more likely to be located within 500 nt upstream and 50 nt downstream of the start codon compared to *cre* sites that were near genes not impacted by CcpA inactivation (*p* < .001 by Fisher's exact test) (Figure 6a). Relative to our previously described in silico derived *cre* (Figure 6e, (DebRoy et al., 2016)), the major difference for our ChIP‐seq derived *cre* was a lack of predominance of T at positions 1 and 2 (Figure 6b). We compared the *cre* sites that we found using the ChIP method in this study to those that we had previously predicted in MGAS2221 using in silico analysis of RNAseq data (DebRoy et al., 2016). Both studies were conducted on mid‐exponential cells grown in THY. We had predicted 72 *cre* sites in silico compared to the 57 we found by ChIP‐seq in this study. Of these sites, only 29 were in common with 20 being transcriptionally altered upon CcpA inactivation in both studies. Importantly, the ChIP‐seq analysis enabled identification of 14 additional binding sites that are near genes that have altered transcript levels in a ∆*ccpA* mutant but were not predicted by the in silico approach. These include the *cre* sites identified in the promoter of *prtS*, *sagA* and the three *cre* sites in the PTS and ABC transport systems for maltose/maltodextrin. Conversely, the in silico method predicted 23 DNA loci that were transcriptionally impacted by CcpA inactivation in our study but were not bound by CcpA in our ChIP‐seq analysis. These genes included those encoding the transcriptional regulator FruR, an alcohol dehydrogenase, and the ABC transporter Spy1310.

When considering sites enriched in 2221‐CcpA‐V301A only (Figure 6c), we identified a motif that was highly similar to that for strain MGAS2221. The occurrence of an A at position 15 was reduced in the *cre* consensus derived from DNA loci in strain 2221‐CcpA‐V301A compared to that derived from MGAS2221. Of 57 *cre* sites in MGAS2221, 37 were bound in strain 2221‐CcpA‐V301, and we did not identify any enriched *cre* containing sites that were present in strain 2221‐CcpA‐V301 but not in MGAS2221. For *cre* sites that were bound only in strain MGAS2221 but not in the strain 2221‐CcpA‐V301A (Figure 6d), the occurrence of a T at position 2 was absolute and markedly increased relative to all *cre* sites in MGAS2221. Thus, the ChIP‐seq data show that although GAS *cre* sites have a similar architecture to other Gram‐positive bacteria, in silico prediction of GAS CcpA binding using motifs is problematic.

### 2.7 Impact of CcpAV301A mutation on magnitude of transcript level variation and degree of DNA binding

Given our finding of the differential transcript level impact of the CcpAV301A change (Figure 3e), we sought to further study how interruption of CcpA‐HPr~P interaction impacted CcpA function by assessing whether the CcpAV301A amino acid variation quantitatively impacted CcpA gene regulation and DNA binding. For clarity of analysis, we only examined genes whose transcript levels were significantly increased by CcpA inactivation in MGAS2221 (e.g., were CcpA repressed), that evidenced in vivo DNA binding by CcpA, and were monocistronic or the first gene in an operon. We identified 33 genes that met these criteria, all but one of which (*argR2*) was associated with a *cre* (Table S2). Using these genes, we tested the hypothesis that the CcpAV301A polymorphism would result in less release of catabolite repression compared with total CcpA inactivation. Indeed, for 27/33 genes, transcript levels were significantly higher in strain 2221∆*ccpA* relative to 2221‐CcpA‐V301A, up to 57‐fold but with a broad range (Table S2).

**FIGURE 7:**
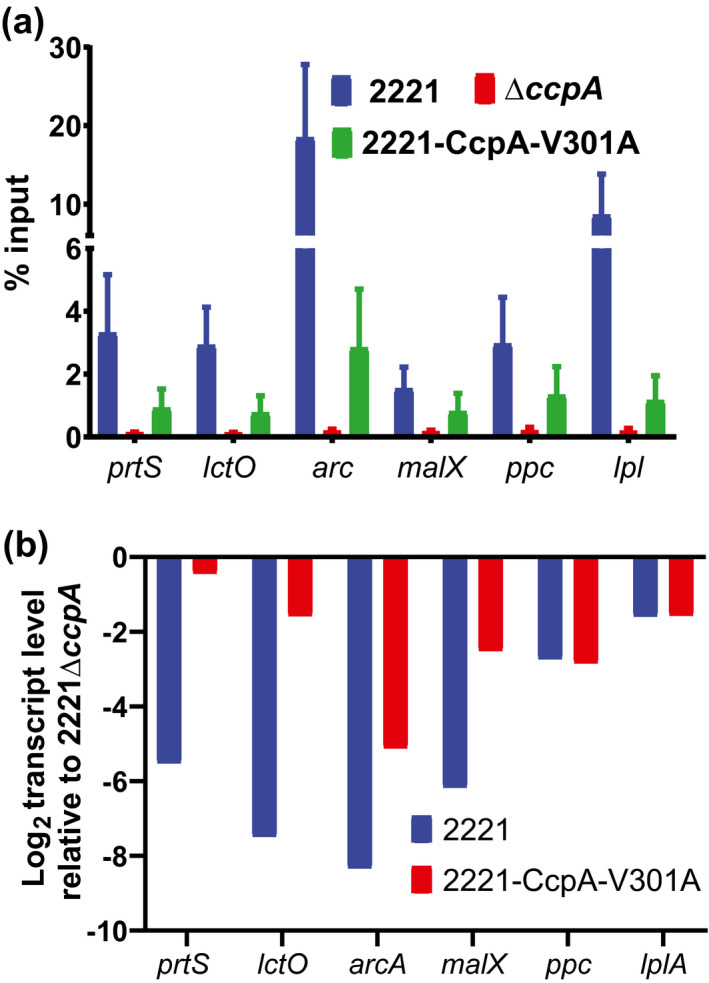
Quantitative impact of CcpAV301A alteration on gene transcript level and DNA binding. (a) SYBR qRT PCR analysis of DNA precipitated using anti‐CcpA antibody from indicated strains (legend inset) for specific promoters (indicated on x‐axis). (b) Transcript level changes as reported in our RNAseq data for the six genes in panel a

Next, we sought to determine whether this differential impact on transcript levels correlated with a change in DNA binding affinity as determined by amount of DNA immunoprecipitated using anti‐CcpA antibody. We used SYBR qPCR to quantify precipitated DNA from the promoter regions of six genes that evidenced CcpA binding for both MGAS2221 and 2221‐V301A‐CcpA strains by ChIP‐seq. Location of predicted *cre* sites in the enriched CcpA binding sites identified in the promoters of these six genes are shown in Figure 5a,b and in Figure S4. For all six genes, we observed significantly more DNA precipitation in MGAS2221 compared to 2221‐CcpA‐V301A and compared to strains 2221∆*ccpA* (Figure 7a). However, we did not identify a consistent relationship between quantitative differences in the amount of DNA precipitated and the gene transcript level variation observed in our RNAseq results (Figure 7b). For example, there was markedly more DNA from the *ppc* and *lplA* promoters precipitated in strain MGAS2221 compared to 2221‐CcpA‐V301A (Figure 7a), yet, there was no significant difference in *ppc* and *lplA* gene transcript levels between strains 2221 and 2221‐CcpA‐V301A relative to 2221∆*ccpA* (Figure 7b). Conversely, there was only a modest difference in the amount of *malX* promoter DNA precipitated from MGAS2221 compared to 2221‐CcpA‐V301A (Figure 7a), yet, we observed marked variation in transcript levels between MGAS2221 and 2221‐CcpA‐V301A relative to 2221Δ*ccpA* (Figure 7b). Taken together, we conclude that abrogation of CcpA‐HPr~P interaction quantitatively impacts CcpA function both in terms of the effect of CcpA on gene transcript levels and DNA binding affinity, but decreases in CcpA affinity for promoter DNA due to the V301A alteration does not strictly correlate with a subsequent impact on gene transcript level under the studied conditions.

## 3 DISCUSSION

The complex interplay between basic metabolic processes and bacterial pathophysiology is being increasingly appreciated (Eisenreich et al., 2010; Pacheco et al., 2012; Rohmer et al., 2011). For Gram‐positive bacteria, CcpA stands squarely at this interface given its central role in controlling preferred carbon utilization pathways and regulating virulence factors in a diverse array of human pathogens (Iyer et al., 2005; Mendez et al., 2012; Shelburne et al., 2008; Vega et al., 2016). The primary link of CcpA to the energy status of the bacterial cell is via its interaction with HPr, but study of HPr in many important Gram‐positive bacteria has been limited due to its essential nature (Fleming et al., 2015; Willenborg et al., 2014). Herein, we sought to extend our knowledge about how CcpA links metabolic and virulence processes through a comprehensive identification of in vivo CcpA binding sites in the major human pathogen group A *Streptococcus*. Additionally, we created a strain in which CcpA cannot interact with HPr~P, to delineate HPr~P‐dependent and HPr~P‐independent aspects of CcpA function. Our findings show that CcpA directly regulates the genes encoding several critical GAS virulence factors and that >50% of genes in the GAS CcpA transcriptome are impacted by CcpA independent of HPr~P.

A key contribution of our study is the first genome wide analysis of in vivo CcpA DNA binding for a β‐hemolytic streptococci. We identified 76 CcpA binding sites in GAS, the specificity of which were demonstrated by the lack of enrichment in a CcpA knockout strain. The only other available CcpA ChIP‐seq analysis in streptococci was performed in *Streptococcus suis* (Willenborg et al., 2014) and identified 58 DNA loci bound by CcpA at the mid‐exponential phase of growth as was studied herein. Some loci, such as those in the promoters of *glpK*, *malX,* and *pgmA* were identified in both studies, while others like *arcA, manLMN, pgi,* and *eno* were not. In MGAS2221 we found the *arcA* site to be the most strongly enriched peak and the most derepressed gene upon *ccpA* deletion. In contrast, the *S. suis* study did not identify enrichment of the *arcA* or the *manLMN* promoters even though these genes were impacted by CcpA inactivation. Additionally, we did not identify the *cre*2 motif present in the *S. suis* data set, perhaps because that motif was identified at the stationary phase of growth, which we did not investigate.

A common observation of both this and the *S. suis* study (Willenborg et al., 2014), and of CcpA investigations in other pathogens (Antunes et al., 2012), is that direct CcpA binding accounts for only a small percentage of genes affected by CcpA inactivation. For example, of the genes encoding the 14 known carbohydrate PTS systems in MGAS2221, the transcript levels of 13 were affected by CcpA inactivation, but only four are directly regulated. This narrow direct impact of CcpA on the PTS systems was also observed in *S. suis*, where CcpA directly impacted only two of the 14 PTS systems present. The *manLMN* operon in GAS and *S. pneumoniae* has been shown to impact a diverse array of other carbohydrate transporters (Abranches et al., 2003, 2006; Fleming & Camilli, 2016; Vadeboncoeur & Pelletier, 1997). We herein established that in GAS, CcpA binds the promoter of the *manLMN* operon in vivo (Table 3), and thus, could indirectly account for some of the broad impact of CcpA inactivation on carbohydrate transport systems despite only directly binding a small number of genes encoding these systems. While it has been postulated that the broad CcpA transcriptome may be due to the impact of CcpA on other regulators (Antunes et al., 2012; Carvalho et al., 2011; DebRoy et al., 2016; Seidl et al., 2009), we found that CcpA only bound the promoters of a small number of other regulator encoding genes (Table 4) which is not consistent with a broad effect of CcpA on the GAS regulatory network. The marked increase in HPr~P observed following CcpA inactivation may account for a substantial proportion of the indirect effects of CcpA given the known role of HPr~P in a broad array of regulatory processes (Deutscher et al., 2005). There has been increasing evidence of the role of sRNAs in gene regulation (Dutta & Srivastava, 2018). While sRNAs have been identified to impact GAS virulence, there is no direct evidence of the impact of sRNAs on metabolic pathways or on any of the highly regulated CcpA targets (Danger et al., 2015; Pappesch et al., 2017; Perez et al., 2009). However, CcpA‐regulated sRNAs have been identified in *S. aureus* and it remains formally possible that sRNAs might mediate some of the regulation observed in GAS upon CcpA inactivation (Bronesky et al., 2019).

Another key finding of this work was our identification that CcpA directly binds to genes encoding three critical GAS virulence factors, *sagA*, *prtS*, and *speB*. How CcpA mechanistically impacts the expression of the *sag* operon, and subsequent production of the key cytotoxin streptolysin S, has been an object of study for the past 12 years since the original observation that CcpA inactivation strikingly increases streptolysin S production (Kinkel & McIver, 2008; Shelburne et al., 2008). Our group as well as others have identified in vitro binding of CcpA to the *sagA* promoter (Kinkel & McIver, 2008; Shelburne et al., 2008) whereas an in vivo study showed no precipitation of the *sagA* promoter using a CcpA antibody (Kietzman & Caparon, 2010). Although both MGAS2221 and HSC5, the strain used in the aforementioned study (Kietzman & Caparon, 2010), both contain the *sagA cre* site we identified, the area upstream of the *cre* site is quite divergent for the two strains which could explain the differential in vivo binding results. It has been shown that the *manLMN* operon and the β‐glucoside PTS (Table 3), which we identified herein as being directly and indirectly regulated by CcpA, respectively, impacts streptolysin production (Braza et al., 2020; Sundar et al., 2017) suggesting that CcpA may regulate streptolysin S production both directly and indirectly. In concert with our data, CcpA was identified as binding in vivo to the gene encoding suilysin, the pore‐forming toxin of *S. suis* (Willenborg et al., 2014) and to bind in vitro to the *sagA* promoter of *S. anginosus* (Bauer et al., 2018). We identified direct binding of CcpA to *speB*, in accordance with previous studies that have used DNA pulldown and fluorescence polarization methods to demonstrate this interaction (Kietzman & Caparon, 2010; Shelburne et al., 2010). Interestingly, *speB* was one of the very few genes directly activated by CcpA. Additionally, our identification that CcpA directly regulates *prtS*, which encodes a critical IL‐8 degrading enzyme, marks the first identification of in vivo binding of a regulator to this important GAS virulence factor. The evolutionary rationale for having such critical and diverse virulence factor encoding genes under direct control of a global metabolic regulator is not clear, but CcpA has also been shown to directly and indirectly impact the expression of key virulence factors in a range of Gram‐positive bacteria from clostridia to staphylococci (Abranches et al., 2008; Chiang et al., 2011; Seidl et al., 2006; Varga et al., 2004).

Although HPr has long been recognized as critical to CcpA function, study of HPr in major human pathogens such as staphylococci and streptococci has been limited due to its essential nature, including the critical Ser46 residue (Fleming et al., 2015; Willenborg et al., 2014). We also were unable to affect HPrSer46~P through a mutagenesis approach, and thus, sought to interrupt CcpA‐HPr~P interaction by modifying the critical CcpAV301 residue. Surprisingly, although the V301A mutation abrogated CcpA‐HPr~P interaction both in vitro and in vivo, more than half of the genes in the CcpA transcriptome still demonstrated CcpA‐based repression in strain 2221‐CcpA‐V301A, indicating the capacity of CcpA to function independently of HPrSer46~P in GAS. We were also able to correlate the preserved function of the CcpAV301A protein through ChIP‐seq analysis which revealed continued enrichment for numerous DNA promoters in strain 2221‐CcpA‐V301A. Our identification of CcpA functioning independent of HPr~P echoes findings from a recent ChIP‐seq study in *S. suis* which found that CcpA can bind to promoters of and regulate several genes in both exponential and stationary phase of growth (Willenborg et al., 2014). Given the absence of HPrSer46~P in the stationary phase, the authors postulated that the CcpA binding and regulation observed in the stationary phase probably occurred independent of HPr~P (Willenborg et al., 2014). These findings stand in contrast to those from *Staphylococcus xylosus* in which HPrSer46~P is absolutely essential for CcpA‐mediated catabolite repression (Jankovic & Bruckner, 2002). Our findings are more in concert with studies in *B. subtilis*, the nonpathogenic gram positive model bacterium, in which the impact of an HPrS46A mutation is essentially an overall dampening of gene regulation in terms of magnitude (Lorca et al., 2005). Indeed, our ChIP‐seq analysis revealed that enrichment of promoter DNA was typically reduced in strain 2221‐CcpA‐V301A compared to the wild‐type even when significant changes in transcript levels were identified, suggesting that the CcpAV301A bound to *cre* sites with lower affinity. Similarly, we found that the magnitude of effect on gene transcript levels due to the inability of the CcpAV301A mutant protein to bind HPrSer46~P varied such that genes could be grouped into classes that are strongly, mildly or not impacted by the abrogation of CcpA‐HPr~P interaction.

One possible purpose of the varied magnitude of effects on gene transcript levels observed in the 2221‐CcpA‐V301A strain would be to facilitate a broad array of transcriptional responses to carbohydrate availability and intracellular energy status (Paluscio et al., 2018). GAS infects numerous different infection sites such as skin, oropharynx, blood, and muscle (Cole et al., 2011; Cunningham, 2000; Walker et al., 2014). Each of these sites have unique nutritional and environmental makeup. The dependence/independence of CcpA from HPr~P could assist with fine‐tuning regulation of pertinent genes by CcpA such that site specific resources can be utilized optimally to aid the pathogen. For example, a recent study compared the GAS transcriptome during necrotizing fasciitis to growth in standard laboratory medium (Kachroo et al., 2020). Some of the most highly upregulated genes in Kachroo et al. including *sagA, arcA*, and the cellobiose PTS operon, were directly bound by CcpA in our study, all of which we classified as HPr~P‐dependent. Conversely, the transcript levels of the directly CcpA bound, HPr~P‐independent genes of the sialic acid operon, *prtS*, *ackA*, and *hpr* showed no significant upregulation during necrotizing fasciitis (Kachroo et al., 2020). These findings are consistent with the concept that CcpA can maintain repression of these genes even under metabolically unfavorable conditions when HPrSer46~P levels would be expected to be low.

In conclusion, herein, we present a genome wide analysis of chromatin occupancy by CcpA which reveals that CcpA directly regulates multiple key GAS virulence factors in addition to a broad array of critical metabolic genes. Through use of a CcpA isoform incapable of interacting with HPr~P, we have delineated not only the role of HPr in CcpA‐mediated gene regulation but also the ability of CcpA to function at certain sites independently of its key co‐factor. These findings extend the mechanistic understanding of how CcpA contributes to the pathophysiology of Gram‐positive bacteria.

## 4 EXPERIMENTAL PROCEDURES

### 4.1 Bacterial strains, media, and growth conditions

All strains used in this study are listed in Table 1. Group A *Streptococcus* (GAS) strains were routinely grown in Todd‐Hewitt media supplemented with 0.2% yeast extract (THY) at 37°C with 5% CO_2_. A single amino acid change in *ccpA* was engineered into the wild type MGAS2221 strain by homologous recombination using the pBBL740 plasmid as described previously (Horstmann et al., 2018), to construct the isoallelic strain 2221‐CcpA‐V301A.

### 4.2 Recombinant proteins and antibodies

Site directed mutagenesis (primers in Table S3) was used to introduce the V301A mutation into an existing clone of GAS *ccpA* (Shelburne et al., 2008) in the pET‐His2 vector to generate the pET‐His2‐V301A plasmid, which was transformed into *E. coli* BL21/pLysS. Wild type CcpA and V301A mutant protein were induced overnight at 18°C, and purified to >95% homogeneity as described previously (Shelburne et al., 2010) and extensively buffer exchanged to 20 mM Tris/HCl pH 7.5, 200 mM NaCl. Recombinant GAS HPr was overexpressed, phosphorylated, and purified as described previously (Shelburne et al., 2010). For the generation of polyclonal antibodies purified wild type CcpA and HPr proteins were used to immunize rabbits and affinity‐purified antibody was obtained (Covance, Denver, PA).

### 4.3 Protein‐protein interaction analysis

Surface Plasmon Resonance (SPR) analysis was performed at 25°C using BIAcore T200 instrument (GE Healthcare). Wild type CcpA and V301A mutant proteins were immobilized on sensor chip CM5 via amine coupling. PBS (phosphate‐buffered saline [8.06 mM Na2HPO4 and 1.94 mM KH2PO4, pH 7.4, 2.7 mM KCl, 137 mM NaCl]) was used as running buffer for immobilization and binding experiment. Sensor chip surface was activated by injecting a 1:1 mixture of 0.4 M of 1‐ethyl‐3‐(3‐dimethylaminopropyl) carbodiimide hydrochloride and 0.1 M of N‐hydroxysuccinimide over the flow cell surface at 5 µl/min for 7 min. After the surface was activated, CcpA (10 µg/ml in 10 mM NaOAc pH 5.0) and CcpAV301A (20 µg/ml in 10 mM NaOAc pH 5.0) were injected over different flow cells. Sensor surface was deactivated with an injection of 1.0 M ethanolamine‐HCl pH 8.5 at 5 µl/min for 7 min. Approximately, 3,700 response units (RU) of CcpA and 4200 RU of CcpAV301A were immobilized. A reference flow cell was prepared with activation and deactivation steps but no protein was immobilized. Two‐fold dilutions of HPr or HPrSer46~P from 1.25 to 80 µM in PBS were flown over the immobilized proteins at 20 µl/min for 30 s. All SPR responses were baseline corrected by subtracting the response generated from the corresponding reference surface. Double‐referenced SPR response curves (with the buffer blank run further subtracted) were used for affinity determination. The equilibrium response of each injection was collected and plotted against the concentration of injected protein. A one‐site binding (hyperbola) model was fitted to the data (GraphPad Prism 4) to obtain the equilibrium dissociation constant *K*⁠_D_.

### 4.4 PhosTag gel

Recombinant HPr protein was phosphorylated and purified as described previously (Shelburne et al., 2010). Cell lysates of GAS strains were prepared, separated on 12.5% SuperSep Phos‐tag gels and detected using a polyclonal anti‐HPr antibody (Horstmann et al., 2014) as described previously (Horstmann et al., 2018). Experiments were repeated at least twice using samples collected on separate days.

### 4.5 Co‐immunoprecipitation

Strains MGAS2221, 2221Δ*ccpA*, and 2221‐CcpA‐V301A were grown to mid‐exponential phase in THY, cross‐linked with EGS [ethylene glycol bis(succinic acid *N*‐hydroxysuccinimide ester)] and formaldehyde and harvested. Pellets were sonicated and CcpA‐containing complexes were immunoprecipitated using a polyclonal anti‐CcpA antibody, and then, analyzed for the presence of HPr by western blotting using a polyclonal anti‐HPr antibody and the Odyssey imaging system as described previously (Horstmann et al., 2015).

### 4.6 Analysis of transcript levels

For RNA seq analysis, RNA was isolated from four replicate cultures for each strain grown to mid‐exponential phase in THY (OD ~ 0.5) using the RNeasy kit (Qiagen) and processed as previously described (Horstmann et al., 2014). RNAseq data analysis was performed as previously described (Sanson & Flores, 2020). As the MGAS2221 genome is not publicly available, we used the MGAS5005 genome as the reference genome, which is identical to MGAS2221 (Sumby et al., 2005) in gene content. MA plots comparing log_2_ fold‐change and transcripts per kilobase million (TPM) were generated for individual comparisons of the wild‐type (MGAS2221) and the isoallelic strain 2221‐CcpA‐V301A to the ∆*ccpA* deletion strain. For each strain, TPM values from replicate samples for each gene within the reference MGAS5005 genome were averaged. Generated plots in combination with differential gene expression values produced by CLC Genomics Workbench (version 20) were used to identify targets with significantly altered transcript levels in individual comparisons. For Taqman real‐time qRT‐PCR, strains were grown in duplicate on two separate occasions to mid‐exponential phase in THY and processed as described previously (Horstmann et al., 2014). The gene transcript levels between MGAS2221 and either 2221∆ *ccpA* or 2221‐CcpA‐V301A were compared using an ordinary one way ANOVA. Primers and probes used are listed in Table S3.

### 4.7 Chromatin immunoprecipitation (ChIP) and sequencing (ChIP‐seq)

For ChIP analysis, MGAS2221, 2221∆*ccpA,* and 2221‐CcpA‐V301A strains were grown to mid‐exponential phase in THY. Crosslinking was performed with 1% formaldehyde and quenched with 0.125 M glycine. DNA was sheared using a Diagenode Bioruptor Plus and immunoprecipitated with anti‐CcpA antibody. We performed ChIP‐seq for two independent replicates of each strain. ChIP sequencing was performed in the Advanced Technology Genomics Core (ATGC) at MD Anderson Cancer Center. Briefly, Illumina compatible Indexed libraries were prepared from 12 ng of Diagenode Bioruptor Pico sheared ChIP DNA using the KAPA Hyper Library Preparation Kit (Roche). Libraries were enriched with 2 cycles of PCR then assessed for size distribution using the 4200 TapeStation High Sensitivity D1000 ScreenTape (Agilent Technologies) and quantified using the Qubit dsDNA HS Assay Kit (Thermo Fisher). Equimolar quantities of the indexed libraries were multiplexed, 9 libraries per pool. The pool was quantified by qPCR using the KAPA Library Quantification Kit (Roche) then sequenced on the Illumina NextSeq500 sequencer using the 75 nt high‐output flow cell. Raw FASTQ files from DNA sequencing were trimmed through Trimmomatic v. 0.36 (Bolger et al., 2014). A sliding window quality trimming was performed, cutting once the average quality of a window of three bases fell <30. Reads shorter than 32 bp after trimming were discarded. Resulting data were aligned to GenBank reference genome of *Streptococcus pyogenes* MGAS5005 (NC_007297.2) using Bowtie2 v. 2.3.0 (Langmead & Salzberg, 2012), without allowing mismatch. Resulting SAM files were converted to BED format using sam2bed v. 2.4.33 from BEDOPS (Neph et al., 2012). In order to identify peaks from Chip‐seq data, we used MACS2 v. 2.1.1.2 (Zhang et al., 2008). First, we estimated the coverage for each base using coverageBed v. 2.27.1 from BEDTools (Quinlan & Hall, 2010). Next, we compared data from the wild type MGAS2221 and 2221‐CcpA‐V301A mutant to the ∆*ccpA* deletion strain (which serves as the control) to remove sequencing noise background based on a coverage ≤2 of fold change. Finally, we applied MACS2 on the clean data using a cutoff q‐value of 0.05 with nonmodel option and MFOLD of “2 50.” The effective genome size was set to 1,838,562. The sequences of assembled peaks were retrieved and submitted to Multiple Em for Motif Elicitation v. 5.0.0 (MEME) (Bailey et al., 2015) for motif searching. The expected number of sites was set to one per sequence and the minimum motif width was set to 15 bp (Sharma et al., 2017). A single statistically significant motif (E‐value < 1e‐12) was recovered for MGAS2221 and 2221‐CcpA‐V301A and these matched the known CcpA binding consensus (DebRoy et al., 2016). The E‐value is derived by MEME, from the motif's log likelihood ratio, taking the motif length and background DNA sequence into account. Manual visualization of identified motifs and closest coding genes (CDS) on *S. pyogenes* MGAS5005 genome was performed using Integrative Genomic Viewer v. 2.3 (IGV) (Thorvaldsdottir et al., 2013).

To verify selected sites of enrichment observed by ChIP‐seq, ChIP DNA was analyzed by SYBR qRT PCR. Primers (Table S3) were designed to flank the respective enriched sites. Each reaction contained 1 µl of ChIP or input DNA, 10 mM primers and 5 µl of PowerUp SYBR Green Master Mix (Applied Biosystems). Reaction were amplified as per manufacturer's instructions followed by a melting curve of the product.

## CONFLICT OF INTEREST

The authors have no conflict of interests.

## AUTHOR CONTRIBUTIONS

S.D., S.A.S., V.A., G.G., M.L., and M.H. designed the research, S.D., S.A., N.H., and S.A.S. did the experiments, S.D., X.L., S.A.S., A.R.F, V.A., G.G., V.M., M.L., and M.H. performed data analysis and S.D., X.L., M.L., M.H., and S.A.S. wrote the manuscript. All authors reviewed the manuscript.

## Supporting information

Fig S1‐S4

Table S1

Table S2

Table S3

## Data Availability

The data that support the findings of this study will be shared upon publication. RNA‐Seq and Chip‐seq data will be deposited at the sequence‐read archive.
