# Supplementary material for: Genome‐wide analysis of in vivo CcpA binding with and without its key co‐factor HPr in the major human pathogen group A Streptococcus
Source: Mol Microbiol. 2020 Dec 29;115(6):1207–28. doi: 10.1111/mmi.14667 (PMC8359418; doi:10.1111/mmi.14667)
Supplement: Supplementary file 1 — Fig S1‐S4 [file MMI-115-1207-s003.pdf]

Supplementary Figures

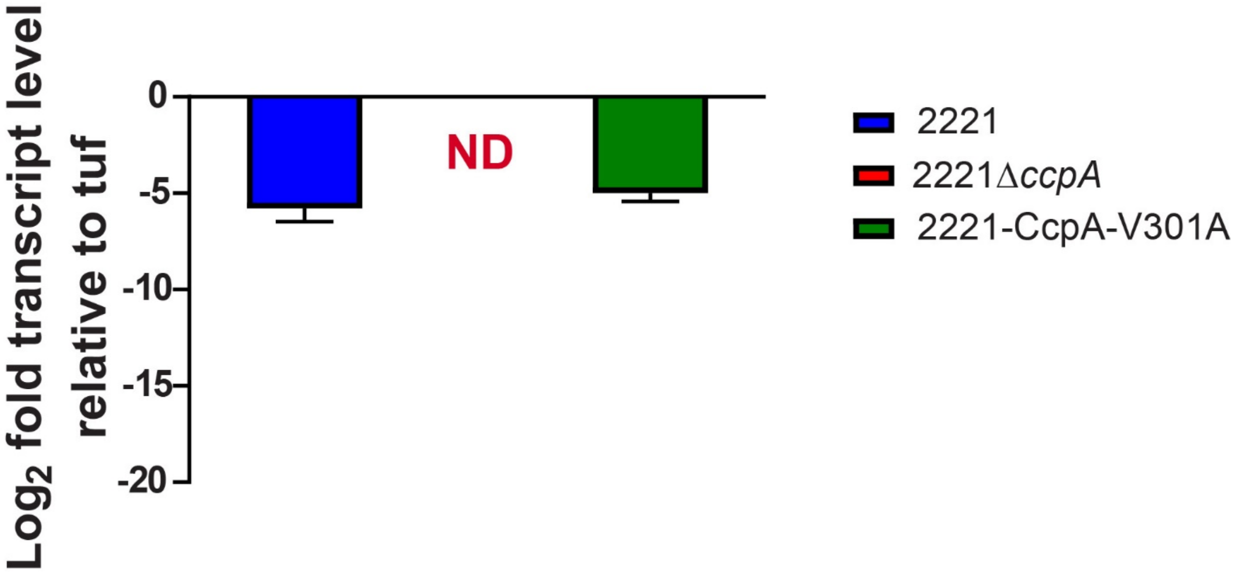

Supplementary Figure S1. Transcript levels of *ccpA* in different GAS strains. Gene transcript levels of *ccpA* were analysed by targeted Taqman qRT-PCR analysis in strains listed in the legend. Data are mean ± standard deviation of two biological replicates, with two technical replicates, done on two separate days (n = 8).

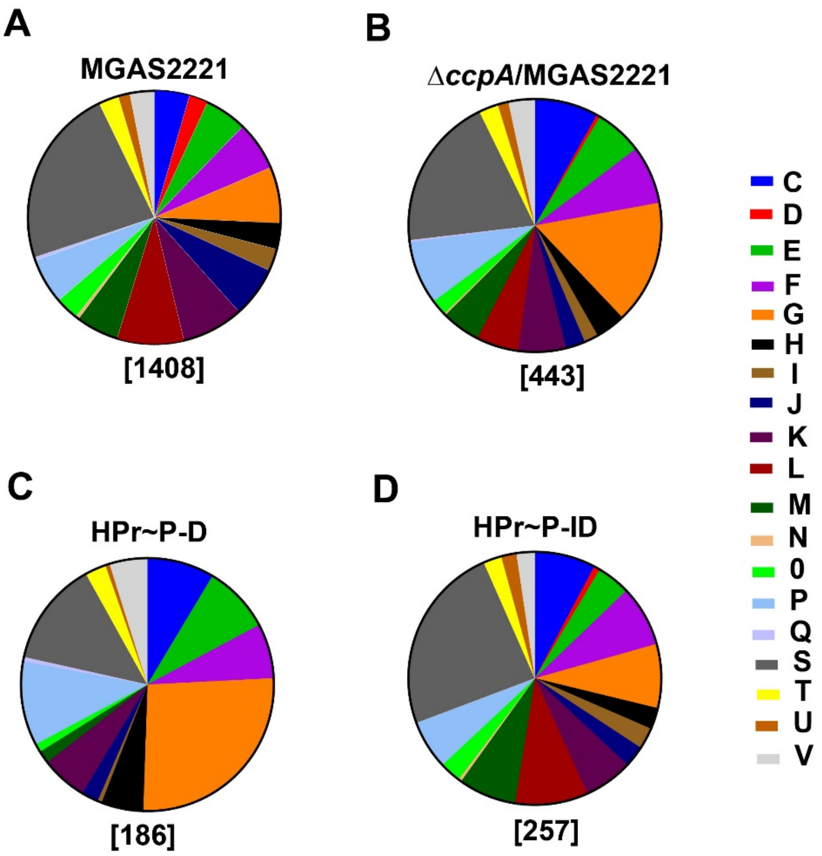

Supplementary Figure S2. COGS analysis of differentially regulated GAS genes. Pie charts depicting COG-based distribution of (A) all genes in MGAS2221, (B) genes impacted by *ccpA* deletion (Δ*ccpA*/MGAS2221) and the (C) HPr~P-dependent (HPr~P-D) and (D) HPr~P-independent (HPr~P-ID) genes. [C] Energy production and conversion; [D] Cell cycle control, cell division, chromosome partitioning; [E] Amino acid transport and metabolism; [F] Nucleotide transport and metabolism; [G] Carbohydrate transport and metabolism; [H] Coenzyme transport and metabolism; [I] Lipid transport and metabolism; [J] Translation, ribosomal structure and biogenesis; [K] Transcription; [L] Replication, recombination and repair; [M] Cell wall/membrane/envelope biogenesis; [N] Cell motility; [O] Posttranslational modification, protein turnover, chaperones; [P] Inorganic ion transport and metabolism; [Q] Secondary metabolites biosynthesis, transport, and catabolism; [S] Function unknown; [T] Signal transduction mechanisms; [U] Intracellular trafficking, se*cre*tion, and vesicular transport and [V].Defense mechanisms.ie charts

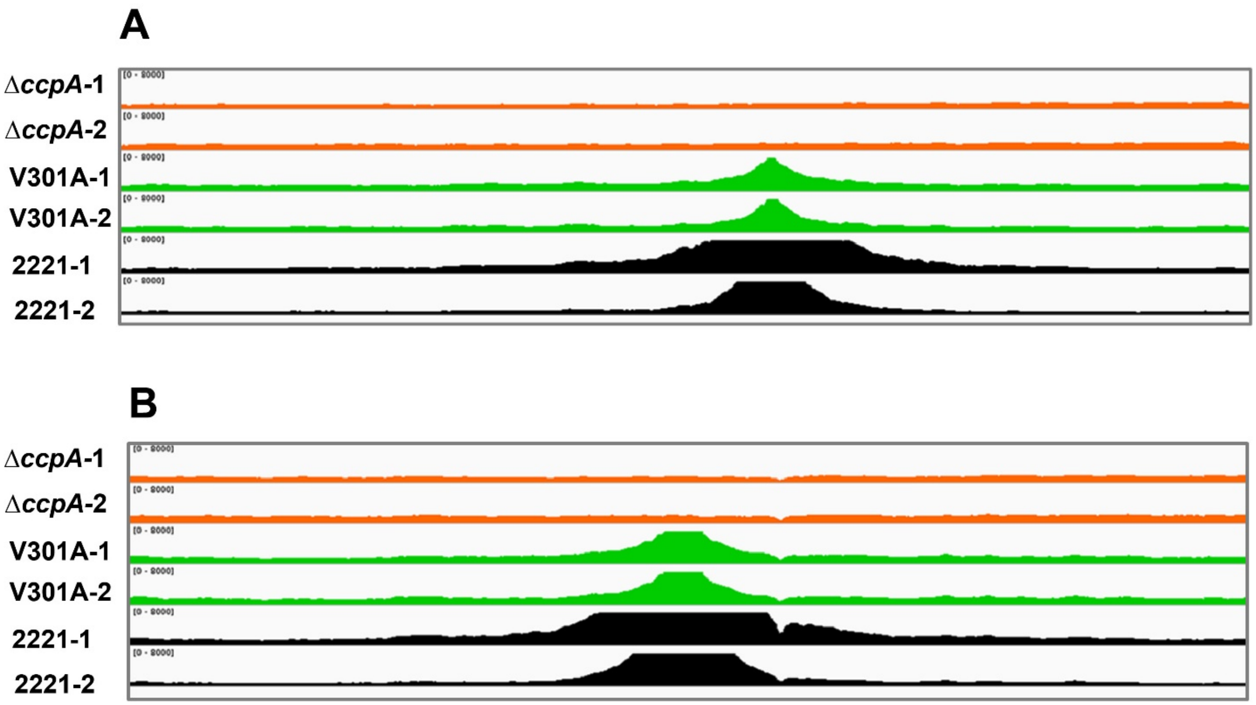

Supplementary Figure S3. Enrichment of CcpA-DNA interaction at select sites. Peaks depicting enrichment of CcpA-DNA binding in the promoters of (A) *arcA* and (B) *phaB* are shown.

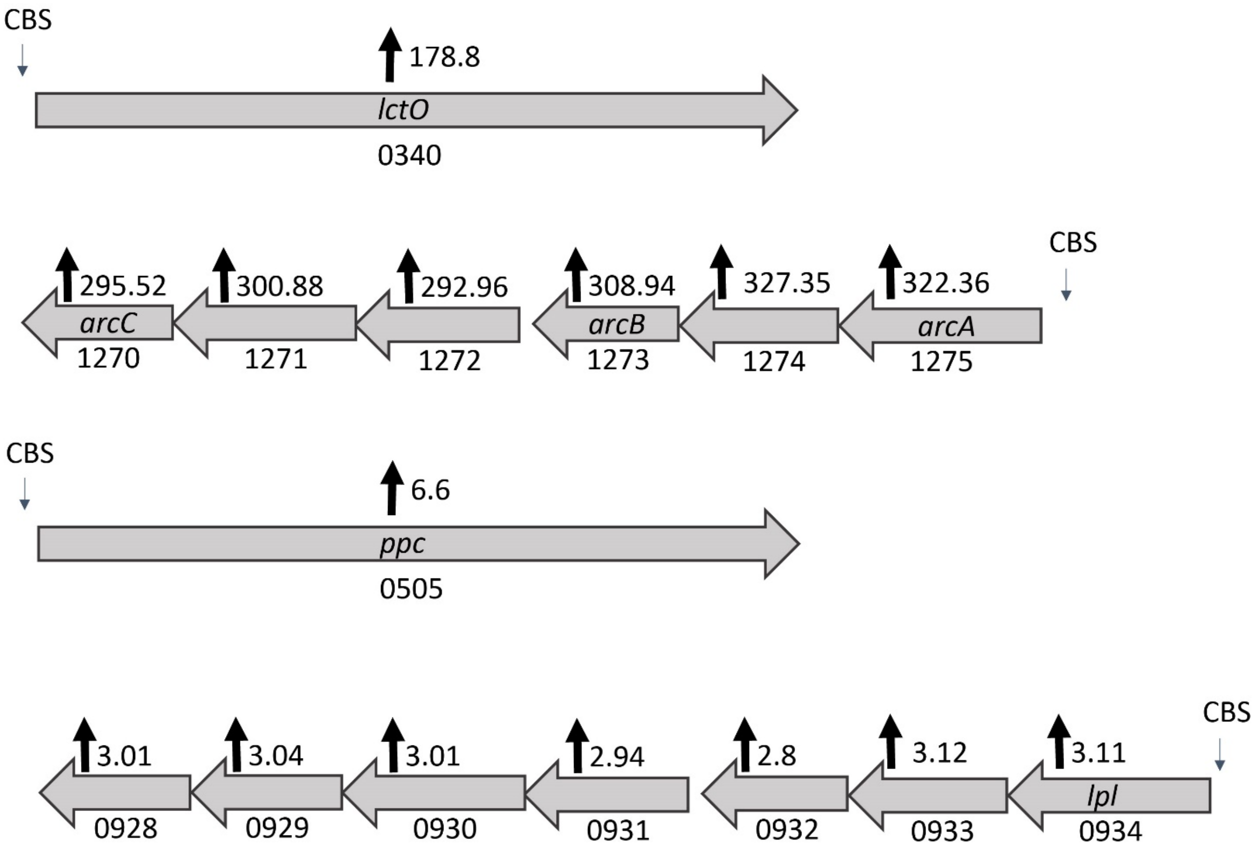

Supplementary Figure S4. CcpA-bound sites in MGAS2221. Schematic representation of four of the six binding sites for CcpA (CBS) enriched in the ChIP-seq experiment and validated by SYBR qRT-PCR. A similar schematic for the remaining two sites is presented in Figure 5. Fold change in transcript levels upon CcpA inactivation is displayed with black arrows. Positive numbers indicate higher transcript levels in 2221Δ*ccpA* compared to MGAS2221.
